# Graph Topology-Guided Multi-Task Learning for Fall Detection

**DOI:** 10.3390/s26175529

**Published:** 2026-08-31

**Authors:** Xiangtao Zhao, Peilin Jin

**Affiliations:** College of Information Science and Technology (College of Cyber Science and Technology), Shihezi University, Shihezi 832003, China; 20241008192@stu.shzu.edu.cn

**Keywords:** fall detection, multi-task learning, graph topology prior, RT-DETR, occlusion robustness

## Abstract

Fall detection faces challenges of visual occlusions and scale variations in complex multi-person scenarios. To address these issues, this paper proposes MTC-DETR, an end-to-end multi-task collaborative detection framework. After a feature extraction backbone, MTC-DETR builds a Dynamic Scale-Routing and Task-Tuning Encoder to adapt to scale variations, which integrates Deformable Large-Kernel Attention for intra-scale long-range feature enhancement and Dynamic Cross-Scale Fusion and Task-Aware Dispatch for adaptive multi-scale aggregation and task-specific feature calibration. Then, a Topology-Guided Dual-Branch Decoder is designed for object detection and keypoint detection. The Topology-Guided Local–Global Synergistic Attention reconstructs occluded keypoints via multi-hop graph convolutions, and the cross-branch pose prior guides Deformable Cross-Attention sampling in the object detection branch to enhance the localization robustness. Finally, a Homoscedastic Uncertainty Dynamic Joint Loss is introduced to resolve gradient conflicts during multi-task optimization. Experimental results show that MTC-DETR achieves mAP@0.5 scores of 92.7%, 88.9%, and 90.4% on the CAUCAFall, DiverseFall10500, and MT-Fall datasets, respectively. The proposed framework outperforms 14 state-of-the-art methods, proving its robustness and potential for real-world fall detection applications. The model was deployed on the Leju Kuavo 5 Robot. Experimental results from the deployment demonstrate that the model achieves an inference speed of 44.2 FPS (INT8) on an edge computing platform, meeting the real-time requirements for fall detection.

## 1. Introduction

Fall detection is critical in intelligent surveillance, smart elderly care, medical monitoring, and human–computer interaction. With the trend of global population aging, falls have emerged as one of the leading causes of accidental injuries among the elderly. Timely fall alerts and medical responses can reduce the disability and death rates associated with fall incidents [1]. Traditional fall detection systems rely on wearable sensors or environmental sensing devices such as radar and infrared sensors. However, wearable devices often face practical challenges, including user wear compliance and recharging demands [2]. In addition, they are susceptible to background noise interference, and fail to provide intuitive on-site visual information [3]. In contrast, vision-based contactless fall detection technology has become a research focus in both academia and industry.

With the advancement of deep learning, visual fall detection has achieved remarkable progress. The end-to-end object detection models represented by DETR (DEtection TRansformer) [4] introduce global self-attention and bipartite graph matching algorithms to discard post-processing steps such as Non-Maximum Suppression (NMS). Particularly, RT-DETR (Real-Time DETR) [5] further improves the inference speed of Transformer architectures. Relying solely on bounding boxes for object localization and classification is insufficient to capture the key features of falling. In addition, researchers have also incorporated keypoint detection into fall detection tasks [6,7]. But real-world fall detection scenarios are typically characterized by spatial occlusion and scale variations.

The spatial occlusion problem is the core bottleneck constraining detection accuracy [8]. For visual occlusion in multi-person scenes, mutual occlusion often covers key discriminative parts such as the head and shoulders. It damages the holistic appearance features, reducing the distinguishability between fall postures and daily activities. In addition, under fixed surveillance cameras, human bodies at varying distances exhibit scale variations. Distant small-scale falling objects often only contain dozens of pixels, and their weak appearance and joint features are easily submerged in background noise [9]. For close-range large-scale human bodies, local body truncation is prone to occur, and incomplete contour and keypoint information will also interfere with overall posture judgment. But most current fall detection models employ simple multi-layer feature map concatenation (e.g., FPN [10]) when handling cross-scale features. Such static fusion mechanisms cannot dynamically adjust receptive fields according to scale variations, nor can they effectively distinguish valid foreground features from redundant background features.

**Figure 1:**
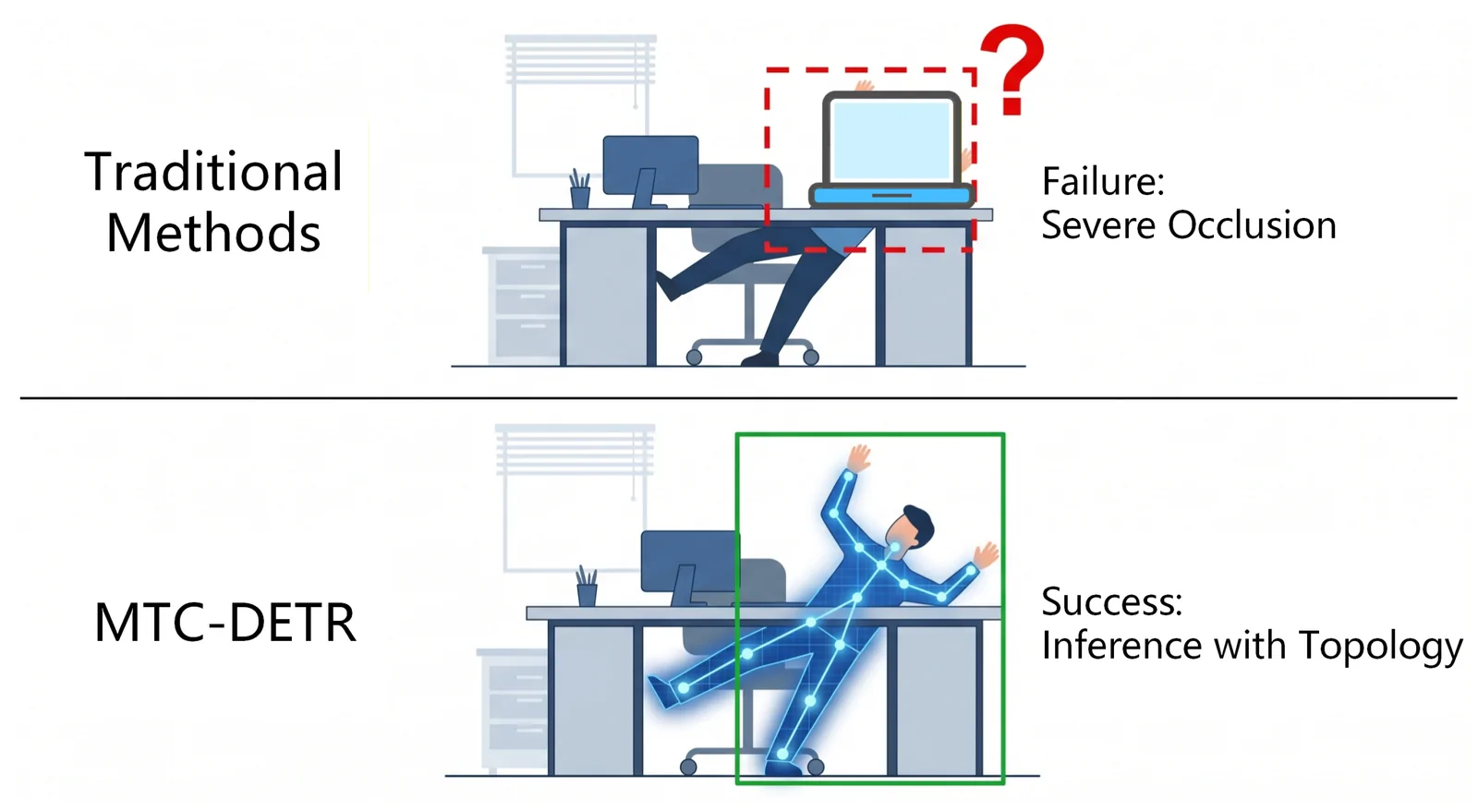
Motivation of the proposed MTC-DETR. Traditional object detectors (**top**) fail to detect the falling people under severe occlusion. In contrast, MTC-DETR (**bottom**) leverages graph topology priors to infer the occluded limbs, achieving robust falling detection.

To address these challenges, particularly the occlusion issue, as illustrated in Figure 1, this paper proposes MTC-DETR (Multi-Task Collaborative DETR), an improved fall detection algorithm that integrates graph topology priors with multi-task learning [11,12]. In this work, multi-task learning specifically denotes an end-to-end parallel dual-task paradigm, which simultaneously performs human fall object detection and keypoint detection without cascaded dependency. Specifically, MTC-DETR constructs a dual-branch multi-task architecture with parallel object detection and keypoint detection branches upon the RT-DETR framework.

Firstly, for scale variations caused by surveillance depth differences, a Dynamic Scale-Routing and Task-Tuning Encoder (DSR-TTE) is designed. The encoder integrates Deformable Large-Kernel Attention (DLKA) [13] for intra-scale long-range feature enhancement, and adopts Dynamic Cross-Scale Fusion to adaptively aggregate hierarchical features across different receptive fields. It further generates two sets of task-specific feature sets for detection and keypoint tasks respectively with Task-Aware Dispatch. Secondly, for visual occlusion in multi-person scenarios, a Topology-Guided Dual-Branch Decoder (TG-DBD) reconstructs occluded features and injects skeletal topology priors into the object detection branch to compensate for the loss of appearance features. The keypoint branch embeds a Topology-Guided Local–Global Synergistic Attention (TG-LGSA) module. Driven by physical constraints of the human skeleton, the module performs topological modeling via graph neural networks [14], and combines local skeletal connectivity with global contextual dependencies to infer occluded structures. Furthermore, a cross-branch pose prior injection mechanism is designed to transfer topological features to the object detection branch, which guides the sampling trajectory of Deformable Cross-Attention and improves detection robustness under severe occlusion. Finally, a Homoscedastic Uncertainty Dynamic Joint Loss (HUDJ-Loss) is introduced for joint optimization [15]. Dual-branch multi-task training often suffers from the “gradient seesaw” effect, where tasks with larger loss values dominate backpropagation. HUDJ-Loss models task-specific observation noise as learnable parameters, and adaptively coordinates optimization gradients across object detection and keypoint detection. Experiments are conducted on three benchmarks: CAUCAFall [16], DiverseFall10500 [17] and the custom MT-Fall dataset. Results show that MTC-DETR consistently outperforms existing state-of-the-art fall detection algorithms.

The main contributions of this paper are summarized as follows:

1. A Dynamic Scale-Routing and Task-Tuning Encoder is proposed to address the scale variation challenge in fall detection. It achieves adaptive multi-scale feature aggregation and task-aware feature calibration, reducing performance degradation caused by scale variance.
2. A Topology-Guided Dual-Branch Decoder is designed to mitigate accuracy loss under occlusion. By embedding graph topology priors and a cross-branch pose prior injection mechanism, it improves detection robustness for both object detection and keypoint detection in occlusion scenarios.
3. A Homoscedastic Uncertainty Dynamic Joint Loss is introduced to resolve gradient conflicts in multi-task optimization. It adaptively coordinates optimization gradients across heterogeneous tasks via learnable observation noise parameters, reducing the “gradient seesaw” effect without manual hyperparameter tuning.
4. Extensive experiments on three benchmarks show that MTC-DETR outperforms 14 state-of-the-art methods, and achieves superior robustness under occlusion and scale variations. And the deployment on the Leju Kuavo 5 Robot demonstrates the effectiveness of the proposed method.

The remainder of this paper is organized as follows. Section 2 reviews related work on vision-based fall detection. Section 3 presents the details of MTC-DETR. Section 4 presents the experimental setup, comparative results, and ablation analysis. Section 5 concludes the paper and discusses future research.

## 2. Related Work

### 2.1. Object Detection-Based Fall Detection

Vision-based fall detection has been widely studied in academia and industry. With the development of deep learning, object detection algorithms play a critical role in this field. Algorithms based on the YOLO series have been introduced and improved due to their balance between detection speed and accuracy. Xu et al. [18] proposed a lightweight algorithm based on YOLOv8n. By introducing the DWR-DRB module and deformable attention mechanism, they enhance the detection capabilities while reducing the parameters and computational complexity. To address the generalization challenges in complex environments, Khan et al. [17] optimized the extraction of spatial and channel features by integrating the Convolutional Block Attention Module (CBAM) in YOLOv8. In specific application scenarios, Nguyen et al. [19] deployed YOLOv7 in an apartment management system to ensure the safety of public areas; Wu et al. [20] proposed a lightweight edge detection system based on the YOLOv11-SEFA to tackle the issues of complex lighting and dense crowds in cultural galleries. To further reduce computational costs, Nabizade et al. [21] designed a lightweight binary fall detection network, LBFD-Net, with depthwise separable convolutions. Regarding the traditional computer vision methods, Kaur et al. [22] explored the effectiveness of combining a hybrid Haar cascade classifier with background subtraction to achieve real-time monitoring on low-cost Raspberry Pi devices.

Despite improvements in accuracy and inference efficiency, such methods essentially rely on bounding boxes for fall detection. When key body parts such as the head and legs are spatially occluded, the lack of discriminative appearance features easily leads to missed detection and false classification. In addition, most existing models adopt static multi-scale fusion architectures, which cannot dynamically adjust receptive fields according to human scale variations at different distances, resulting in performance degradation in scenes with large depth-of-field differences.

### 2.2. Keypoint Detection-Assisted Fall Detection

Although object detection methods have achieved remarkable results, keypoint detection-based methods exhibit robustness. Inturi et al. [23] utilized the AlphaPose network to extract high-quality human keypoints. They conducted deep modeling of the spatial correlations and long-term temporal dependencies of these keypoints by combining CNN and LSTM networks. Addressing complex real-world environments with severe occlusions, such as construction sites, Kim et al. [24] proposed YOSAP-LSTM. This model integrates YOLOv8 for object detection, the SORT algorithm for trajectory tracking, and AlphaPose for posture extraction, achieving exceptional detection specificity and sensitivity on edge computing devices. To tackle the challenges of fall recognition in multi-person scenarios, Liu et al. [25] proposed a hybrid detection method based on an improved YOLOv8s and AlphaPose, which enhances small object feature extraction via SPD-Conv. In addition, to overcome the model generalization degradation caused by cross-camera viewpoints, Dutra et al. [26] leveraged a virtual engine to generate 3D synthetic data. Combined with geometric data augmentation techniques, they enhanced the cross-domain generalization capability of 2D pose models under unseen camera perspectives.

However, most existing keypoint detection-based fall detection schemes adopt a cascaded pipeline of “object detection first, then keypoint estimation”. The accuracy of keypoint detection is directly limited by upstream detection results, and errors accumulate layer by layer with the increase in crowd density.

### 2.3. Temporal Modeling for Fall Detection

Since a fall is a spatiotemporal dynamic process with contextual coherence, temporal motion modeling has emerged as another research direction in vision-based fall detection. Some studies utilize time-series models to capture complex actions. Raza et al. [27] and Aydoğan et al. [28] introduced Vision Transformer [29] into the processing of keypoint features, which utilizes multi-head attention to learn and distinguish the deep spatiotemporal differences between normal human behaviors and fall events. Sanjalawe et al. [30] proposed a hybrid deep learning system integrating YOLOv8 with a Time-Space Transformer. This system not only achieves high-precision real-time spatial localization but also filters out false alarms caused by activities in daily living. Cho et al. [31] proposed a hybrid model based on Dynamic Graph Neural Networks (DGNNs) and LSTM, which decouples motion prediction from gait classification. It can not only identify falls that have already occurred but also achieve anticipatory fall detection when the human body is in a transient state of imbalance. Meanwhile, the MMEFD framework proposed by Ros et al. [32] tracks the motion trajectory within a specific time window, providing a flexible temporal solution for multi-view video analysis that breaks away from static preset thresholds.

Although temporal modeling can capture the dynamic process of falls, such methods rely on continuous video frame input, which increases computational overhead. More importantly, temporal modeling only supplements motion information, and cannot fundamentally solve the spatial feature loss caused by occlusion, so it still faces performance bottlenecks in severely occluded scenes.

### 2.4. Multi-Modal Fall Detection

To broaden the application of vision-based fall detection, some researchers have investigated in imaging modalities to overcome the limitations of traditional visible-light cameras. Yang et al. [33] extended the input source of visual detection to infrared videos. By combining a fine-tuned keypoint detection network with a two-stream Spatial-Temporal Graph Convolutional Network (ST-GCN), they achieved robust action recognition in both thermal and near-infrared imagery. For highly privacy-sensitive environments, Mobsite et al. [34] employed monocular depth cameras to extract Global History of Motion (GHM) images and depth frame sequences. Through keypoint analysis using a lightweight network and a dilated ConvLSTM, they accomplished high-accuracy fall determination without exposing users’ facial or color features. Yu et al. [35] proposed a disjointed representation network, DisJRNet. This model learns to separate human and background components directly at the feature maps, enabling greater focus on the fall-related motion itself when facing complex environments.

But multi-modal schemes rely on additional sensing hardware such as infrared cameras and depth cameras, which greatly increase deployment cost and maintenance difficulty.

### 2.5. Research Gaps and Motivation

Despite considerable progress in vision-based fall detection, existing methods still suffer from performance degradation in real-world complex scenarios. Single-task object detectors are vulnerable to severe performance degradation under spatial occlusion, whereas cascaded keypoint detection pipelines suffer from accumulated errors that rise linearly with crowd density. Meanwhile, static multi-scale fusion structures cannot dynamically adapt to scale variations. To address these issues, this paper proposes MTC-DETR based on RT-DETR, with improvements in scale-adaptive encoding, topology-guided decoding and dynamic multi-task loss balancing.

## 3. Materials and Methods

### 3.1. Overall Architecture

**Figure 2:**
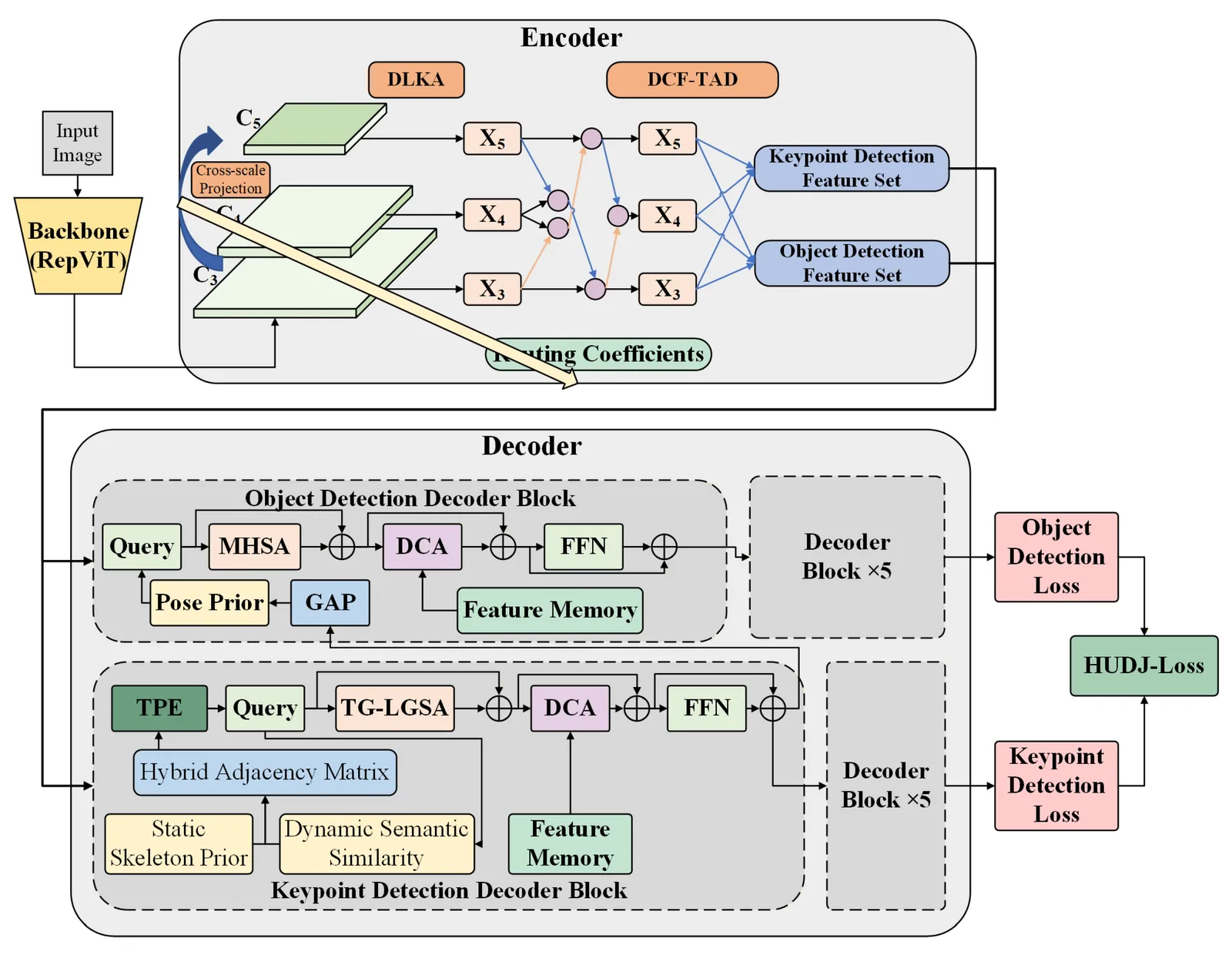
The overall architecture of the proposed MTC-DETR framework.

As illustrated in Figure 2, the proposed MTC-DETR is an end-to-end Transformer-based multi-task collaborative framework tailored for occluded fall detection. Notably, MTC-DETR performs detection based on single-frame images and cannot analyze the complete dynamic process of falling actions, resulting in inherent limitations in distinguishing falls from highly similar static postures such as voluntary lying down. In contrast, this single-frame paradigm achieves low-latency, low-computation inference without relying on consecutive video frames, showing better adaptability to resource-constrained edge deployment scenarios.

The multi-task mechanism consists of two parallel and collaborative sub-tasks: one is the object detection task for human localization and fall state classification, and the other is the keypoint detection task for human skeleton structure extraction. The two tasks share the feature extraction backbone and interact bidirectionally during the decoding stage, rather than adopting a unidirectional cascaded pipeline, and they jointly support accurate fall posture discrimination.

Built on the above dual-task collaborative mechanism, the entire framework comprises three core components: a lightweight multi-scale feature extraction backbone, a Dynamic Scale-Routing and Task-Tuning Encoder (DSR-TTE), and a Topology-Guided Dual-Branch Decoder (TG-DBD), which are jointly optimized end-to-end via a Homoscedastic Uncertainty Dynamic Joint Loss (HUDJ-Loss).

First, the RepViT-M2.3 [36] backbone extracts a set of hierarchical multi-scale features $\left\{C_{3},C_{4},C_{5}\right\}$ from the input image, with features at different levels capturing semantic information of varying granularity. These features are then fed into the DSR-TTE encoder, which replaces the original AIFI and CCFM modules in RT-DETR. The encoder consists of two sub-modules. Deformable Large-Kernel Attention (DLKA)-based Intra-Scale Long-Range Feature Enhancement module is performed on the $C_{5}$ feature to model long-range spatial dependencies with linear computational complexity. On this basis, Dynamic Cross-Scale Fusion and Task-Aware Dispatch (DCF-TAD) performs adaptive cross-scale feature aggregation through a data-driven dynamic routing mechanism, and further recalibrates the enhanced feature pyramid. This step yields two task-specific Key/Value feature sets: ${\tilde{F}}_{det}$ optimized for object detection, and ${\tilde{F}}_{kpt}$ optimized for keypoint detection.

During the multi-task decoding phase, in contrast to the single-branch detection decoder adopted in RT-DETR and RT-DETRv4, MTC-DETR initializes two sets of learnable queries and processes them through a parallel dual-branch architecture with layer-wise cross-task collaborative interaction. In the keypoint detection branch, keypoint queries are fused with physical skeletal priors and dynamic semantic correlations. Then, the Topology-Guided Local–Global Synergistic Attention (TG-LGSA) module, a newly introduced graph topology modeling component absent in the original RT-DETR decoder, reconstructs occluded keypoints with Graph Convolutional Networks (GCNs). The refined topological features from the keypoint branch are compressed into a pose prior embedding via global average pooling after each decoding layer. This layer-wise pose prior injection mechanism, another extension beyond the RT-DETR framework, guides the sampling trajectories of Deformable Cross-Attention (DCA) in the detection branch as a structural navigator.

Finally, different from the standalone detection loss used in the RT-DETR series, the HUDJ-Loss is introduced to coordinate the optimization gradients across the object detection and keypoint detection tasks. By modeling the observation noise of each task as learnable homoscedastic uncertainty, the loss function adaptively adjusts the weight of each task according to its convergence state, eliminating the gradient seesaw effect and ensuring stable global convergence.

### 3.2. Dynamic Scale-Routing and Task-Tuning Encoder

The original RT-DETR encoder consists of two components: the Attention-Based Intra-Scale Feature Interaction (AIFI) module operating on the highest-level feature map $C_{5}$ for global dependency modeling, and the Cross-Scale Feature-Fusion Module (CCFM) built upon fixed convolution and up-sampling for multi-scale aggregation. This static architecture limits the ability to capture cross-scale deformations in fall events. To address this limitation, the Dynamic Scale-Routing and Task-Tuning Encoder (DSR-TTE) replaces both AIFI and CCFM. Taking multi-scale backbone features $\left\{C_{3},C_{4},C_{5}\right\}$ as input, the encoder performs feature enhancement and aggregation through two sub-modules, namely, Deformable Large-Kernel Attention (DLKA) and Dynamic Cross-Scale Fusion and Task-Aware Dispatch (DCF-TAD).

#### 3.2.1. Deformable Large-Kernel Attention-Based Intra-Scale Long-Range Feature Enhancement

In the original RT-DETR, the AIFI module flattens the 2D feature map into a 1D token sequence and models long-range dependencies through a standard Transformer with multi-head self-attention. While effective for extracting high-level semantics, the single-scale $C_{5}$ feature lacks fine-grained spatial details from low-level layers and standard MHSA brings precision loss when facing crowds.

Although the original DLKA [13] can effectively model long-range spatial dependencies, it still has limitations when directly applied to multi-scale fall detection scenarios. First, it performs attention computation independently on each single-scale feature map, lacking cross-level information interaction. Thus, high-level semantic features cannot obtain fine-grained spatial details from low-level layers, leading to weak feature representation for small-scale distant fall objects. Second, if low-level features are directly fused without constraints, background noise will be introduced, which aggravates the interference of crowded complex backgrounds on occluded fall target localization. To address these issues, a Cross-Scale Projection (CSP) mechanism is introduced with learnable gated fusion based on the basic DLKA structure. It projects low-level high-resolution features to the high-level feature space, and adaptively controls the fusion proportion through learnable scalar gates, which supplements spatial details for high-level features while suppressing background noise.

Specifically, for the highest-level feature $C_{5}\in R^{H_{5}\times W_{5}\times C_{5}}$, features from $C_{3}$ and $C_{4}$ are projected to the same spatial resolution and channel dimension via down-sampling projection, and fused into $C_{5}$ with learnable scalar gates to avoid overwhelming native semantics. The down-sampling projection function adopts a 3×3 strided depthwise convolution, followed by a 1×1 pointwise convolution for channel alignment. The fused input feature $X_{5}$ for DLKA is formulated as

(1) $$X_{5}=C_{5}+\sum_{j\in \{3,4\}}\sigma \left(\alpha_{j\to 5}\right)\cdot \phi_{j\to 5}\left(C_{j}\right)$$

where σ(·) denotes the sigmoid function, $\alpha_{j\to 5}$ is a learnable scalar gate parameter for the projection path from scale *j* to scale 5, and $\phi_{j\to 5}\left(\cdot \right)$ is the down-sampling projection function, defined as

(2) $$\phi_{j\to 5}\left(C_{j}\right)={\mathrm{Conv}}_{1\times 1}\left({\mathrm{DWConv}}_{3,\;s=2^{5-j}}\left(C_{j}\right)\right)$$

For $C_{3}$ and $C_{4}$, native features are directly used as DLKA inputs, i.e., $X_{3}=C_{3}$, $X_{4}=C_{4}$.

**Figure 3:**
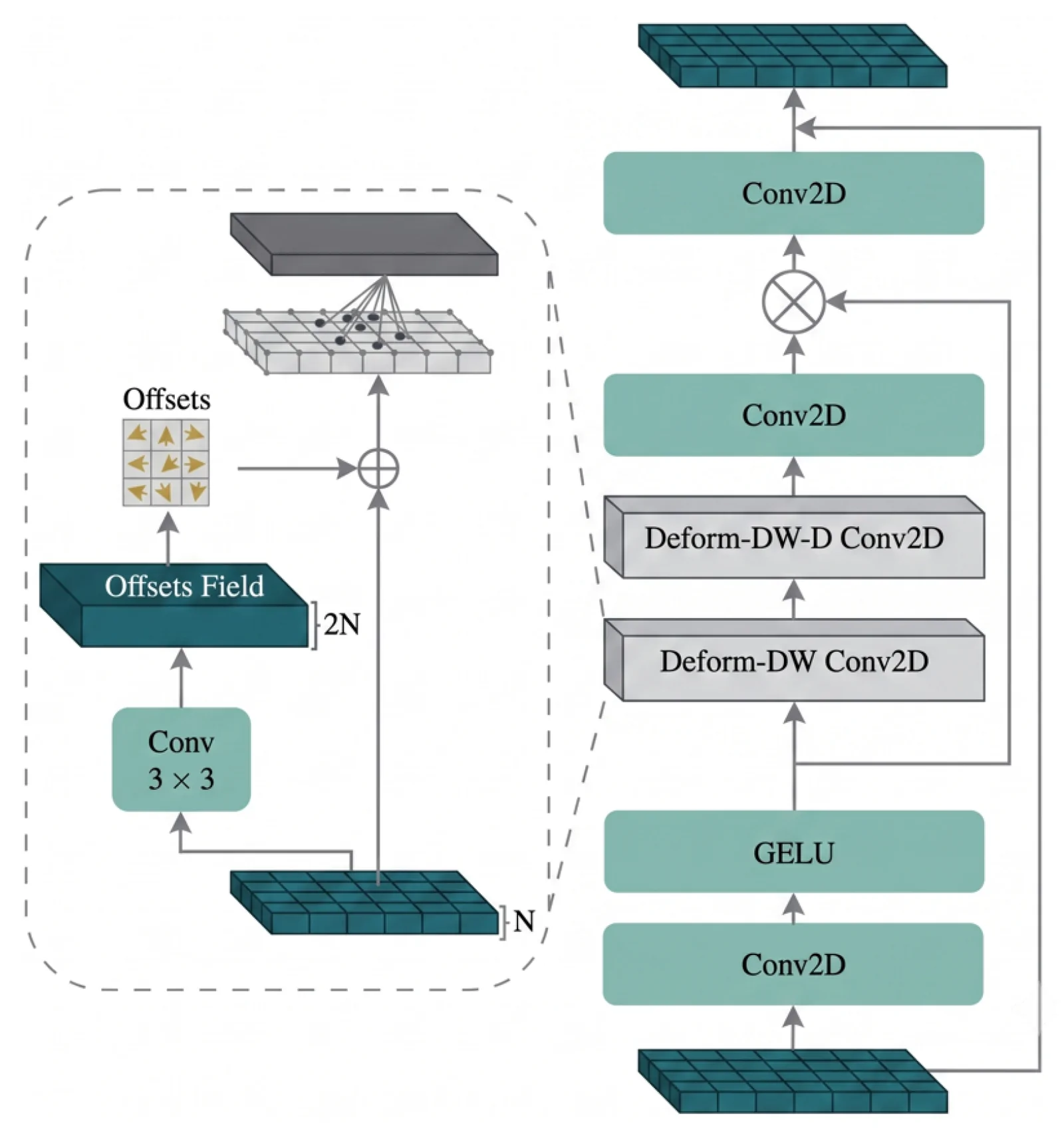
The overall architecture of Deformable Large-Kernel Attention.

As illustrated in Figure 3, DLKA follows the decomposed large-kernel spatial attention paradigm. The basic unit, marked by the dashed box on the left, serves as the core implementation of Deform-DW Conv2D and Deform-DW-D Conv2D. The main module on the right implements the full attention computation.

The basic Deform-DW Conv2D unit takes an *N*-channel feature map as input, which is fed into both the offset prediction branch and the feature sampling branch simultaneously. In the offset prediction branch, the input feature first passes through a 3 × 3 Conv2D layer to output a 2N-channel Offsets Field. Each sampling point requires offset prediction in both *x* and *y* spatial directions, so the number of channels is twice that of the input. The offsets field is further decoded into coordinate offsets ΔP for each sampling point, and a parallel prediction branch outputs the per-position attention modulation tensor ΔM to control the contribution weight of each sampling point. In the sampling and convolution stage, the predicted offsets are superimposed on the original regular sampling grid of the convolution to obtain an adaptively deformed irregular sampling grid. Since the offset values are floating-point numbers, bilinear interpolation is applied to sample feature values at corresponding positions on the input feature map. Finally, Deform-DW Conv2D executes standard depthwise convolution with the number of groups equal to the number of channels, and each channel performs convolution independently. Deform-DW-D Conv2D introduces a dilation coefficient *d* on the basis of depthwise convolution, which further expands the receptive field without increasing the number of parameters to capture longer-range spatial context.

The entire DLKA attention module follows the classic spatial attention paradigm of attention weight generation and original feature weighting. The input feature first undergoes preliminary cross-channel fusion via a 1 × 1 convolution to obtain the preprocessed feature $X_{\mathrm{pre}}$. The feature then passes through two cascaded deformable depthwise convolutions: the first Deform-DW Conv2D extracts local spatial features with a dynamic sampling grid to preliminarily model deformation, producing the local enhanced intermediate feature $F_{\mathrm{ddw}}$; the second Deform-DW-D Conv2D expands the receptive field through dilation to capture long-range spatial context, producing the long-range enhanced intermediate feature $F_{\mathrm{ddwd}}$. The extracted spatial features are processed by the second 1 × 1 convolution to generate a spatial attention weight map Attn, which is multiplied element-wise with the layer-normalized input feature $X_{\mathrm{norm}}$ to perform spatial attention weighting, yielding the attention-weighted feature $X_{\mathrm{weighted}}$. Finally, the third 1 × 1 convolution completes channel fusion and feature calibration to output the final enhanced feature $X_{\mathrm{attn}}$, which can be directly integrated into subsequent network layers.

The complete forward pass of DLKA is summarized in Algorithm 1.
**Algorithm 1** DLKA based Intra-Scale Long-Range Feature Enhancement**Input:** Multi-scale backbone features $C=\{C_{3},C_{4},C_{5}\}$ **Output:** Enhanced features $X=\{X_{3},X_{4},X_{5}\}$
  1:   % Low-level feature projection for the highest-scale feature  2:$X_{5}\leftarrow C_{5}$  3:**for** each scale j∈{3,4} **do**  4:   $X_{5}\leftarrow X_{5}+\sigma \left(\alpha_{j\to 5}\right)\cdot \phi_{j\to 5}\left(C_{j}\right)$    % Gated projection fusion  5:**end for**  6:$X_{3}\leftarrow C_{3}$, $X_{4}\leftarrow C_{4}$  7:   % DLKA enhancement for each scale  8:**for** each scale l∈{3,4,5} **do**  9:   $\mathrm{residual}\leftarrow X_{l}$10:   $X_{\mathrm{norm}}\leftarrow \mathrm{LayerNorm}\left(X_{l}\right)$11:   $X_{\mathrm{pre}}\leftarrow \mathrm{GELU}\left({\mathrm{Conv}}_{\mathrm{pre}}\left(X_{\mathrm{norm}}\right)\right)$12:   $\left\{\Delta P_{1},\Delta M_{1}\right\}\leftarrow {\mathrm{OffsetHead}}_{1}\left(X_{\mathrm{pre}}\right)$    % Predict offsets for Deform-DW Conv2D13:   $F_{\mathrm{ddw}}\leftarrow \text{Deform-DW}\;\mathrm{Conv}2D\left(X_{\mathrm{pre}},\Delta P_{1},\Delta M_{1}\right)$14:   $\left\{\Delta P_{2},\Delta M_{2}\right\}\leftarrow {\mathrm{OffsetHead}}_{2}\left(F_{\mathrm{ddw}}\right)$    % Predict offsets for Deform-DW-D Conv2D15:   $F_{\mathrm{ddwd}}\leftarrow \text{Deform-DW-D}\;\mathrm{Conv}2D\left(F_{\mathrm{ddw}},\Delta P_{2},\Delta M_{2}\right)$16:   $\mathrm{Attn}\leftarrow {\mathrm{Conv}}_{\mathrm{mid}}\left(F_{\mathrm{ddwd}}\right)$    % Generate attention weight map17:   $X_{\mathrm{weighted}}\leftarrow X_{\mathrm{norm}}⊙\mathrm{Attn}$    % Element-wise attention weighting18:   $X_{\mathrm{attn}}\leftarrow {\mathrm{Conv}}_{\mathrm{post}}\left(X_{\mathrm{weighted}}\right)$19:   $X_{l}\leftarrow X_{\mathrm{attn}}+\mathrm{residual}$    % Residual connection20:   $\mathrm{residual}\leftarrow X_{l}$21:   $X_{l}\leftarrow \mathrm{FFN}\left(\mathrm{LayerNorm}\left(X_{l}\right)\right)+\mathrm{residual}$    % FFN with residual connection22:**end for**23:**Return** *X*


#### 3.2.2. Dynamic Cross-Scale Fusion and Task-Aware Dispatch

DCF-TAD improves the cross-scale aggregation paradigm with an input-adaptive interaction mechanism to enhance scale adaptiveness, and appends a task-specific calibration stage to generate differentiated feature representations for the object detection and keypoint detection tasks respectively. The module takes the multi-scale features $X=\{X_{3},X_{4},X_{5}\}$ from the DLKA module as input, and finally outputs two feature sets for downstream decoding branches.

**Figure 4:**
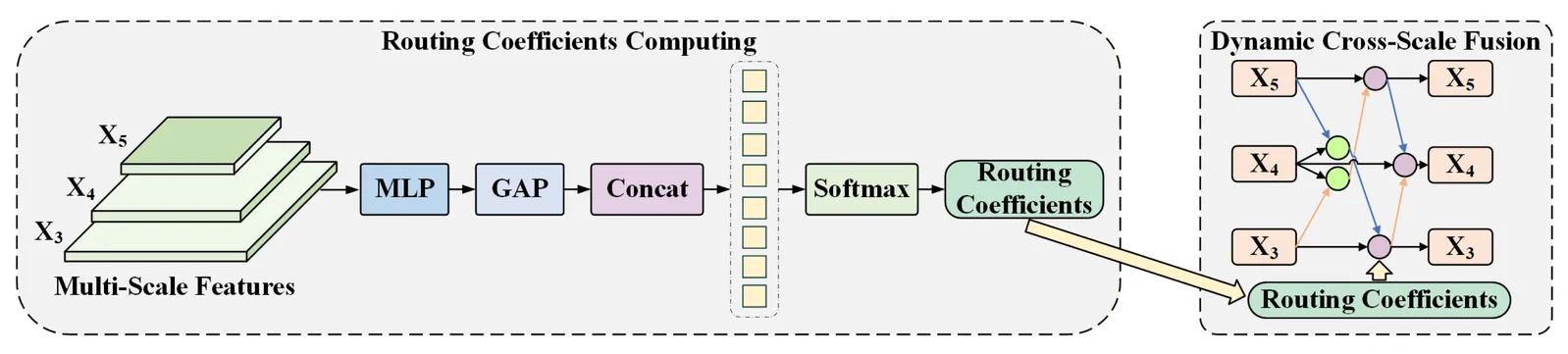
The schematic diagram of the dynamic cross-scale fusion process.

As shown in Figure 4, the fusion process is guided by routing coefficients generated from global scale context. First, global average pooling is performed independently on each scale feature to obtain channel-wise global descriptors, which are concatenated to construct a global scale context vector. The vector is fed into a two-layer lightweight multi-layer perceptron (with dimension identical to the input feature channel and GELU as the activation function) to output raw routing logits, which are divided into three groups corresponding to the three scales.

Each group is normalized via Softmax to produce fusion weights. Specifically, the group for the bottom scale l=3 generates weights $w_{3\_\mathrm{self}}$ and $w_{3\_\mathrm{sem}}$, which control the proportion of native bottom features and top-down semantic supplementary features in the boundary enhancement stage, respectively; the group for the middle scale l=4 generates weights $w_{4\_\mathrm{td}}$ and $w_{4\_\mathrm{bu}}$, which control the contribution ratio of the top-down semantic path and the bottom-up detail path in the reverse aggregation stage; the group for the top scale l=5 generates weights $w_{5\_\mathrm{self}}$ and $w_{5\_\det}$, which control the proportion of native top features and bottom-up detail supplementary features in the boundary enhancement stage, respectively.

The cross-scale fusion is completed in three sequential steps, where the routing weights are dynamically applied to the corresponding fusion operations. First, taking the middle-level feature $X_{4}$ as the core, preliminary fusion is performed with high-level and low-level features separately to generate two sets of intermediate transition features. The top-level feature $X_{5}$ is up-sampled via bilinear interpolation and channel-aligned via a 1×1 convolution. Then, it is element-wise added with $X_{4}$ and calibrated to generate intermediate feature $M_{td}$. Correspondingly, the bottom-level feature $X_{3}$ is down-sampled via a 3×3 depthwise convolution and channel-aligned via a 1×1 convolution, then fused with $X_{4}$ to generate the detail-enhanced intermediate feature $M_{bu}$. Native features of each scale are fully retained at this stage to ensure the stability of basic feature representation. The calculation formulas are as follows:

(3) $$\begin{array}{cc} M_{td} & =T_{4}^{td}\left(X_{4},\;X_{5}\right)\\ M_{bu} & =T_{4}^{bu}\left(X_{4},\;X_{3}\right) \end{array}$$

where $T_{l}^{td}\left(\cdot \right)$ denotes the top-down fusion operation at scale *l*, including up-sampling, channel alignment, element-wise addition and feature calibration; $T_{l}^{bu}\left(\cdot \right)$ denotes the bottom-up fusion operation at scale *l*, including down-sampling, channel alignment, element-wise addition and feature calibration.

On this basis, boundary scale enhancement is performed. The two sets of intermediate features obtained from the first step are fused with the bottom and top scales respectively. The output features of boundary scales are generated with corresponding routing coefficients. For the bottom scale l=3, $M_{td}$ is up-sampled and channel-aligned with the shape of $X_{3}$, then fused with the native $X_{3}$ feature under the weights of $w_{3\_\mathrm{self}}$ and $w_{3\_\mathrm{sem}}$. The fused result is calibrated by a 3×3 depthwise convolution with batch normalization and GELU activation to obtain the final bottom-level output feature ${\tilde{E}}_{3}$. For the top scale l=5, the detail-enhanced feature $M_{bu}$ is down-sampled and channel-aligned to the resolution of $X_{5}$, then fused with the native $X_{5}$ feature with $w_{5\_\mathrm{self}}$ and $w_{5\_\det}$. The final top-level output feature ${\tilde{E}}_{5}$ is obtained after the same convolution. The calculation formulas are as follows:

(4) $$\begin{array}{cc} {\tilde{E}}_{3} & ={\mathrm{Conv}}_{cal}\left(w_{3\_\mathrm{self}}\cdot X_{3}+w_{3\_\mathrm{sem}}\cdot {\mathrm{Proj}}_{up}\left(M_{td}\right)\right)\\ {\tilde{E}}_{5} & ={\mathrm{Conv}}_{cal}\left(w_{5\_\mathrm{self}}\cdot X_{5}+w_{5\_\det}\cdot {\mathrm{Proj}}_{down}\left(M_{bu}\right)\right) \end{array}$$

where ${\mathrm{Proj}}_{up}\left(\cdot \right)$ and ${\mathrm{Proj}}_{down}\left(\cdot \right)$ denote the up-sampling projection and down-sampling projection operations, respectively, and ${\mathrm{Conv}}_{cal}\left(\cdot \right)$ denotes the feature calibration convolution with batch normalization and GELU.

Finally, reverse aggregation for the middle layer is performed. The enhanced boundary features obtained from the second step are fed back to the middle scale to supplement the information of the middle-level hub feature. Specifically, ${\tilde{E}}_{5}$ is up-sampled and channel-aligned to the middle scale, and ${\tilde{E}}_{3}$ is down-sampled and channel-aligned to the middle scale. The two adjusted boundary features are weighted by $w_{4\_\mathrm{td}}$ and $w_{4\_\mathrm{bu}}$ respectively, then added to the native $X_{4}$ feature in a residual manner. The calculation of ${\tilde{E}}_{4}$ is as follows:

(5) $${\tilde{E}}_{4}={\mathrm{Conv}}_{cal}\left(X_{4}+w_{4\_\mathrm{td}}\cdot {\mathrm{Proj}}_{up}\left({\tilde{E}}_{5}\right)+w_{4\_\mathrm{bu}}\cdot {\mathrm{Proj}}_{down}\left({\tilde{E}}_{3}\right)\right)$$

Based on the aggregated feature pyramid $\left\{{\tilde{E}}_{3},{\tilde{E}}_{4},{\tilde{E}}_{5}\right\}$, the task-aware dispatch stage introduces spatial-semantic collaborative reweighting at the encoder–decoder interface to resolve the feature preference mismatch between heterogeneous tasks. Independent channel reweighting modules are constructed and each module aggregates complementary information from two dimensions: the spatial dimension captures fine-grained local edge and texture cues via a 3×3 depthwise convolution, and the semantic dimension extracts global high-level semantic statistics via global average pooling. The outputs of the two modules are concatenated along the channel dimension and projected by a 1×1 convolution. Then, Sigmoid is performed to generate channel-wise attention weights. The task-specific features at each scale are formulated as

(6) $$\left\{\begin{array}{c} {\tilde{F}}_{det,\;l}={\tilde{E}}_{l}⊙\sigma \left(\Phi_{det}\left({\tilde{E}}_{l}\right)\right)\\ {\tilde{F}}_{kpt,\;l}={\tilde{E}}_{l}⊙\sigma \left(\Phi_{kpt}\left({\tilde{E}}_{l}\right)\right) \end{array}\right.$$

where σ is the Sigmoid function and ⊙ denotes channel-wise multiplication. Intuitively, this lightweight channel-wise reweighting delivers effective task differentiation based on the inherent semantic specialization of convolutional channels. Object detection relies primarily on high-level semantic channels encoding holistic human contours and category information, while keypoint detection depends more on fine-grained detail channels capturing local limb edges and texture cues. The learned task-specific attention masks activate task-relevant channels and suppress redundant responses, achieving feature disentanglement between heterogeneous tasks with minimal computational cost. This operation only recalibrates channel response distribution without altering spatial dimensions. The two resulting feature pyramids, denoted as ${\tilde{F}}_{det}=\left\{{\tilde{F}}_{det,3},{\tilde{F}}_{det,4},{\tilde{F}}_{det,5}\right\}$ and ${\tilde{F}}_{kpt}=\left\{{\tilde{F}}_{kpt,3},{\tilde{F}}_{kpt,4},{\tilde{F}}_{kpt,5}\right\}$, serve as the key and value inputs in the detection and keypoint branches respectively.

### 3.3. Topology-Guided Dual-Branch Decoder

The Topology-Guided Dual-Branch Decoder (TG-DBD) includes two parallel branches: the topology-guided keypoint detection branch and the pose-prior-guided object detection branch.

#### 3.3.1. Topology-Guided Keypoint Detection Branch

In the keypoint detection branch, a set of learnable keypoint queries $Q_{kpt}\in R^{N\times K\times d}$ is initialized, where *N* denotes the maximum number of detection objects, K=17 corresponds to 17 human keypoints, and *d* is the feature dimension. Each object is assigned *K* keypoint queries. Each decoder layer takes the task-specific keypoint features ${\tilde{F}}_{kpt}$ as key and value inputs.

As presented in Figure 2, a hybrid adjacency matrix is first constructed before the first decoding layer to provide structural constraints. The matrix fuses the static physical skeleton prior $A_{prior}$ and a sample-adaptive dynamic semantic correlation matrix $A_{dyn}$. $A_{prior}$ encodes fixed human kinematic connections. And $A_{dyn}$ is derived by performing L2 normalization on the current layer’s keypoint query features followed by pairwise similarity calculation, without introducing additional heavy parametric modules. This computation can be parallelized with the decoder’s feature update process, resulting in negligible extra computational latency. During training, $A_{dyn}$ presents dispersed and unstructured correlation distributions in the early training phase, when the model predominantly depends on the static skeleton prior $A_{prior}$ to provide fundamental topological constraints. As training progresses, $A_{dyn}$ gradually converges to stable semantic correlation patterns aligned with human posture dependencies, which captures long-range semantic associations beyond physical skeletal connections. This evolved dynamic prior further regularizes inter-keypoint message passing, and boosts the model’s keypoint reasoning capability and localization accuracy under occlusion and complex posture conditions. The hybrid adjacency matrix is formulated as

(7) $$\tilde{A}={\tilde{D}}^{-\frac{1}{2}}\left(\alpha A_{prior}+\left(1-\alpha \right)A_{dyn}+I\right){\tilde{D}}^{-\frac{1}{2}}$$

where α is a learnable balance factor, *I* denotes the identity matrix for self-loop connections, and $\tilde{D}$ is the degree matrix derived from the fused topology.

Based on the normalized graph Laplacian matrix $L=I-\tilde{A}$, Topological Positional Encoding (TPE) is generated to embed skeletal structural priors into keypoint queries. Different from traditional sin or learnable 2D positional encodings that only model spatial coordinate relationships, TPE exploits the topological connectivity of human keypoints. TPE is computed via per-sample online eigenvalue decomposition to extract low-frequency spectral components, which are further projected to obtain structural position embeddings:

(8) $$E_{topo}=U_{:,2:m+1}W_{topo}$$

where *U* denotes the eigenvector matrix of *L*, *m* is the number of selected non-trivial eigenvalues, and $W_{topo}\in R^{m\times d}$ is a learnable projection matrix. The TPE is added to initial keypoint queries, denoted as ${\hat{H}}^{\left(0\right)}=Q_{kpt}+E_{topo}$.

**Figure 5:**
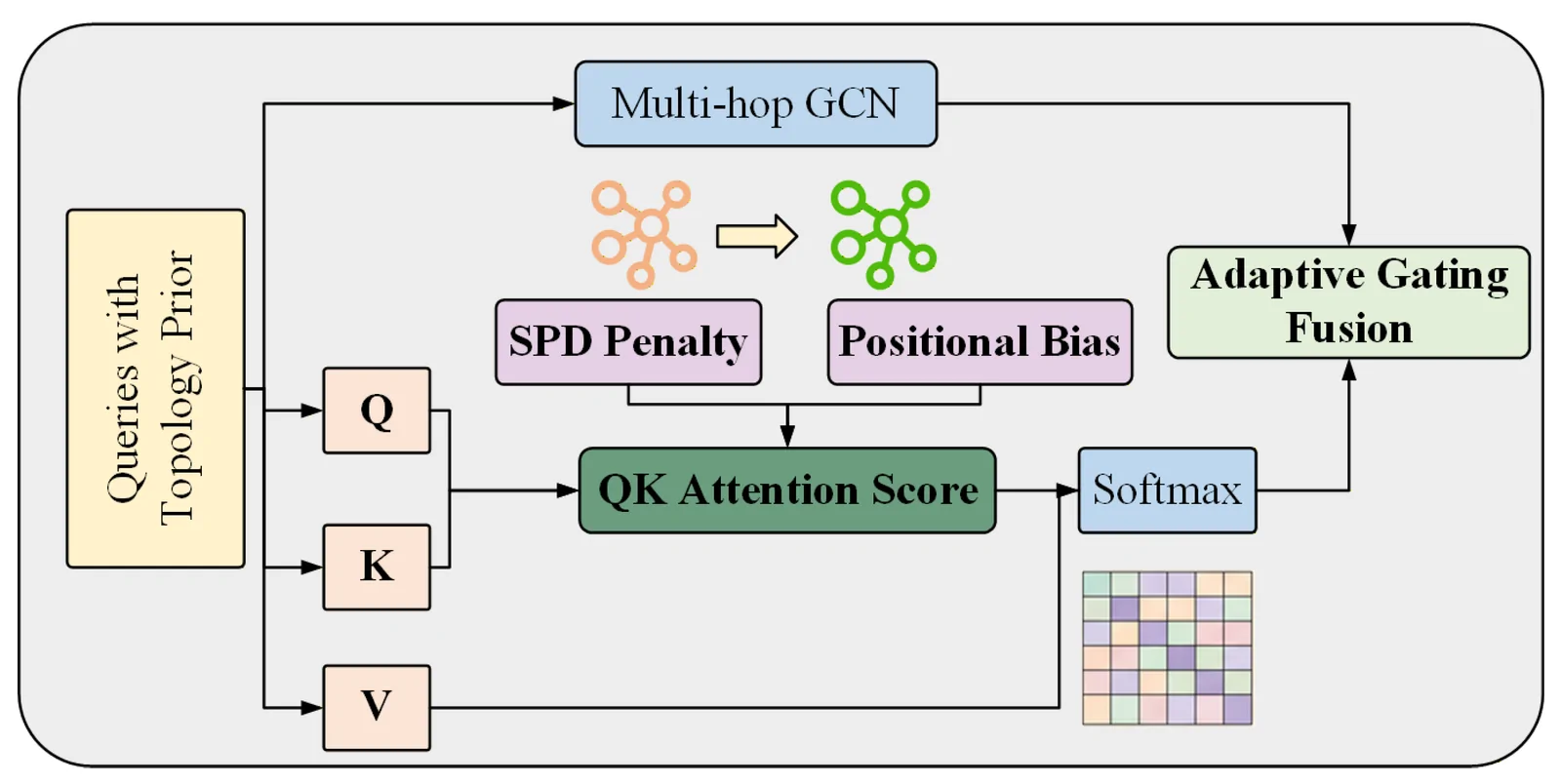
The structure of TG-LGSA module.

To address keypoint occlusion and non-rigid posture deformation during falls, this paper designs the TG-LGSA module, which consists of three parts, as illustrated in Figure 5: multi-hop local topological aggregation, global spatial attention with shortest path distance penalty (SPD), and adaptive gating fusion. TG-LGSA performs intra-query topological relationship reasoning, and serves as the self-attention unit in original RT-DETR decoder layers.

For local topological aggregation, a multi-hop graph convolution is designed. Each node aggregates *S*-hop features to compensate for information loss of occluded keypoints, with S=3 as default. For the *h*-th attention head, the input query features are first projected into value space, and the local feature output is formulated as

(9) $$F_{local,h}=\sigma \left(\sum_{s=0}^{S}\theta_{s,h}{\left({\tilde{A}}^{\left(h\right)}\right)}^{s}\hat{H}W_{V,h,s}\right)$$

where ${\left({\tilde{A}}^{\left(h\right)}\right)}^{s}$ represents the *s*-th-order power of the adjacency matrix for the *h*-th head, $\theta_{s,h}$ is a learnable path decay factor, and $\hat{H}$ denotes the query features of the current layer. Multi-head local features are concatenated to form the final local representation $F_{local}$.

To capture long-range posture dependencies, multi-head global spatial attention with shortest-path distance penalty is introduced. The penalty term reduces interference between different persons in crowded scenes. The input features are first projected into query, key and value spaces, and the global attention output for each head is computed as

(10) $$F_{global,h}=\mathrm{Softmax}\left(\frac{Q_{g,h}K_{g,h}^{T}}{\sqrt{d_{h}}}+B_{rel,h}-\gamma D_{SPD}\right)V_{g,h}$$

where $D_{SPD}\in R^{K\times K}$ is the graph shortest path distance matrix of the human keypoints, γ is a learnable penalty coefficient, and $B_{rel,h}$ denotes the relative spatial positional bias of the *h*-th head. Multi-head outputs are concatenated to form the final global representation $F_{global}$.

An adaptive gating mechanism is adopted to fuse local topological features and global spatial features in a channel-wise manner. It dynamically adjusts the contribution of local constraints and global posture dependencies via a two-layer fully connected MLP with GELU activation, whose input and output channel dimensions match the decoder feature dimension:

(11) $$Z_{self}=M_{gate}⊙F_{local}+\left(1-M_{gate}\right)⊙F_{global}$$

where $M_{gate}=\mathrm{Sigmoid}\left(\mathrm{MLP}\left(F_{local}\|F_{global}\right)\right)$ is the channel-wise gating tensor, and ⊙ denotes element-wise multiplication. The output $Z_{self}$ is the final output of the TG-LGSA module.

As illustrated in Figure 2, the output of TG-LGSA is first added with a residual connection and layer normalization, then fed into Deformable Cross-Attention (DCA) to interact with the task-specific features ${\tilde{F}}_{kpt}$. After another residual connection and layer normalization, a feed-forward network (FFN) generates outputs. The process for the *l*-th decoder layer is

(12) $$\begin{array}{c} {\hat{H}}_{self}^{\left(l\right)}=\mathrm{LayerNorm}\left(Z_{self}^{\left(l\right)}\right)+{\hat{H}}^{\left(l-1\right)}\\ {\hat{H}}_{cross}^{\left(l\right)}=\mathrm{LayerNorm}\left(\mathrm{DefAttn}\left({\hat{H}}_{self}^{\left(l\right)},{\tilde{F}}_{kpt}\right)\right)+{\hat{H}}_{self}^{\left(l\right)}\\ {\hat{H}}^{\left(l\right)}=\mathrm{FFN}\left(\mathrm{LayerNorm}\left({\hat{H}}_{cross}^{\left(l\right)}\right)\right)+{\hat{H}}_{cross}^{\left(l\right)} \end{array}$$

The dynamic semantic correlation matrix is recomputed at each layer based on the updated query features. This recomputation is derived purely from feature similarity without trainable parameters, serving only as a forward structural constraint. Gradients do not backpropagate through the matrix itself, but are transmitted to query features via subsequent graph convolution operations. After *L* layers of iterative decoding, the final queries are mapped to keypoint detection results. The overall workflow of the keypoint detection branch is summarized in Algorithm 2.
**Algorithm 2** Topology-Guided Keypoint Decoder**Input:** Keypoint queries $Q_{kpt}$, task-specific keypoint features ${\tilde{F}}_{kpt}$ **Output:** Joint coordinates, visibility scores
  1:Build initial hybrid adjacency matrix $\tilde{A}$ from skeleton prior and query similarity  2:Compute Topological Positional Encoding $E_{topo}$ from $\tilde{A}$  3:$\hat{H}\leftarrow Q_{kpt}+E_{topo}$  4:**for** each decoder layer l=1→L **do**  5:   Update dynamic semantic matrix $A_{dyn}$ from current $\hat{H}$, refresh $\tilde{A}$  6:   $F_{local}\leftarrow \text{Multi-head}\;\text{Multi-hop}\;\mathrm{GCN}\left(\hat{H},\tilde{A},S\right)$  7:   $F_{global}\leftarrow \text{Multi-head}\;\mathrm{SpatialAttention}\left(\hat{H},D_{SPD}\right)$  8:   $M_{gate}\leftarrow \sigma \left(\mathrm{MLP}\left(F_{local}\|F_{global}\right)\right)$  9:   $Z_{self}\leftarrow M_{gate}⊙F_{local}+\left(1-M_{gate}\right)⊙F_{global}$10:   ${\hat{H}}_{self}\leftarrow \mathrm{LayerNorm}\left(Z_{self}\right)+\hat{H}$11:   ${\hat{H}}_{cross}\leftarrow \mathrm{LayerNorm}\left(\mathrm{DefCrossAttn}\left({\hat{H}}_{self},{\tilde{F}}_{kpt}\right)\right)+{\hat{H}}_{self}$12:   $\hat{H}\leftarrow \mathrm{FFN}\left(\mathrm{LayerNorm}\left({\hat{H}}_{cross}\right)\right)+{\hat{H}}_{cross}$13:**end for**14:Predict keypoints from final $\hat{H}$15:**Return** Keypoints


#### 3.3.2. Pose-Prior-Guided Object Detection Branch

In the cross-branch pose prior injection mechanism, refined keypoint query representations from the keypoint branch are adopted as pose priors to guide the feature sampling process of the detection branch. After each decoding layer of the keypoint branch, global average pooling is performed, compressing per-object keypoint queries into pose prior embeddings. The formulation is given as

(13) $$q_{pose}=\frac{1}{K}\sum_{i=1}^{K}h_{kpt,i}$$

where $h_{kpt,i}$ denotes the updated feature of the *i*-th keypoint query, and $q_{pose}\in R^{N\times d}$ carries skeleton topology information.

As presented in Figure 2, the pose prior embedding is first added to object detection queries before self-attention computation:

(14) $${\tilde{Q}}_{det}=Q_{det}+q_{pose}$$

where $Q_{det}\in R^{N\times d}$ is the query feature from the previous decoding layer.

Following the standard RT-DETR decoder pipeline, the fused queries first pass through a multi-head self-attention (MHSA) module. After residual connection and layer normalization, feature interaction between queries and image features is implemented via Deformable Cross-Attention (DCA). Different from standard cross-attention that computes attention over all spatial positions of the feature map, DCA only performs sparse sampling at a small set of reference points. The fused object queries ${\tilde{Q}}_{det}$ embedded with pose priors are fed into the offset prediction head to generate sampling parameters, and the final attention output is obtained by the weighted aggregation of features at sampling positions:

(15) $$\mathrm{DCA}\left({\tilde{Q}}_{det},{\tilde{F}}_{det}\right)=\sum_{k=1}^{K_{s}}W_{k}\cdot {\tilde{F}}_{det}\left(p_{k}+\Delta p_{k}\right)\cdot \Delta m_{k}$$

$K_{s}$ denotes the total number of sparse sampling points per query; $p_{k}$ is the coordinate of the *k*-th preset regular reference sampling point; $\Delta p_{k}$ is the learnable 2D spatial offset predicted from ${\tilde{Q}}_{det}$; $\Delta m_{k}\in \left(0,1\right)$ is the learnable modulation scalar that controls the importance weight of each sampling point; $W_{k}$ denotes the learnable projection weight for the corresponding sampling point. For occluded regions with missing visual pixels, the model can infer human boundary contours based on the topology prior embedded in the queries, and adjust the sampling trajectory accordingly to reduce localization drift in bounding boxes. Taking the task-specific detection feature ${\tilde{F}}_{det}$ as key and value inputs, the object detection branch finally outputs human bounding box coordinates and fall state classification results after iterative decoding. The pose-prior-guided object detection decoding process is summarized in Algorithm 3.
**Algorithm 3** Pose-Prior-Guided Object Detection Decoding**Input:** Object queries $Q_{det}$, keypoint features $H_{kpt}$, detection features ${\tilde{F}}_{det}$
**Output:** Bounding boxes, fall classification results
  1:**for** each decoder layer l=1→L **do**  2:   $q_{pose}\leftarrow \frac{1}{K}\sum_{i=1}^{K}h_{kpt,i}$  3:   ${\tilde{Q}}_{det}\leftarrow Q_{det}+q_{pose}$  4:   $Q_{self}\leftarrow \mathrm{MHSA}\left(\mathrm{LayerNorm}\left({\tilde{Q}}_{det}\right)\right)+{\tilde{Q}}_{det}$  5:   $Q_{cross}\leftarrow \mathrm{DCA}\left(\mathrm{LayerNorm}\left(Q_{self}\right),{\tilde{F}}_{det}\right)+Q_{self}$  6:   $Q_{det}\leftarrow \mathrm{FFN}\left(\mathrm{LayerNorm}\left(Q_{cross}\right)\right)+Q_{cross}$  7:**end for**  8:Predict boxes and fall class from final $Q_{det}$  9:**Return** bounding boxes, classification results


### 3.4. Homoscedastic Uncertainty Dynamic Joint Loss

**Figure 6:**
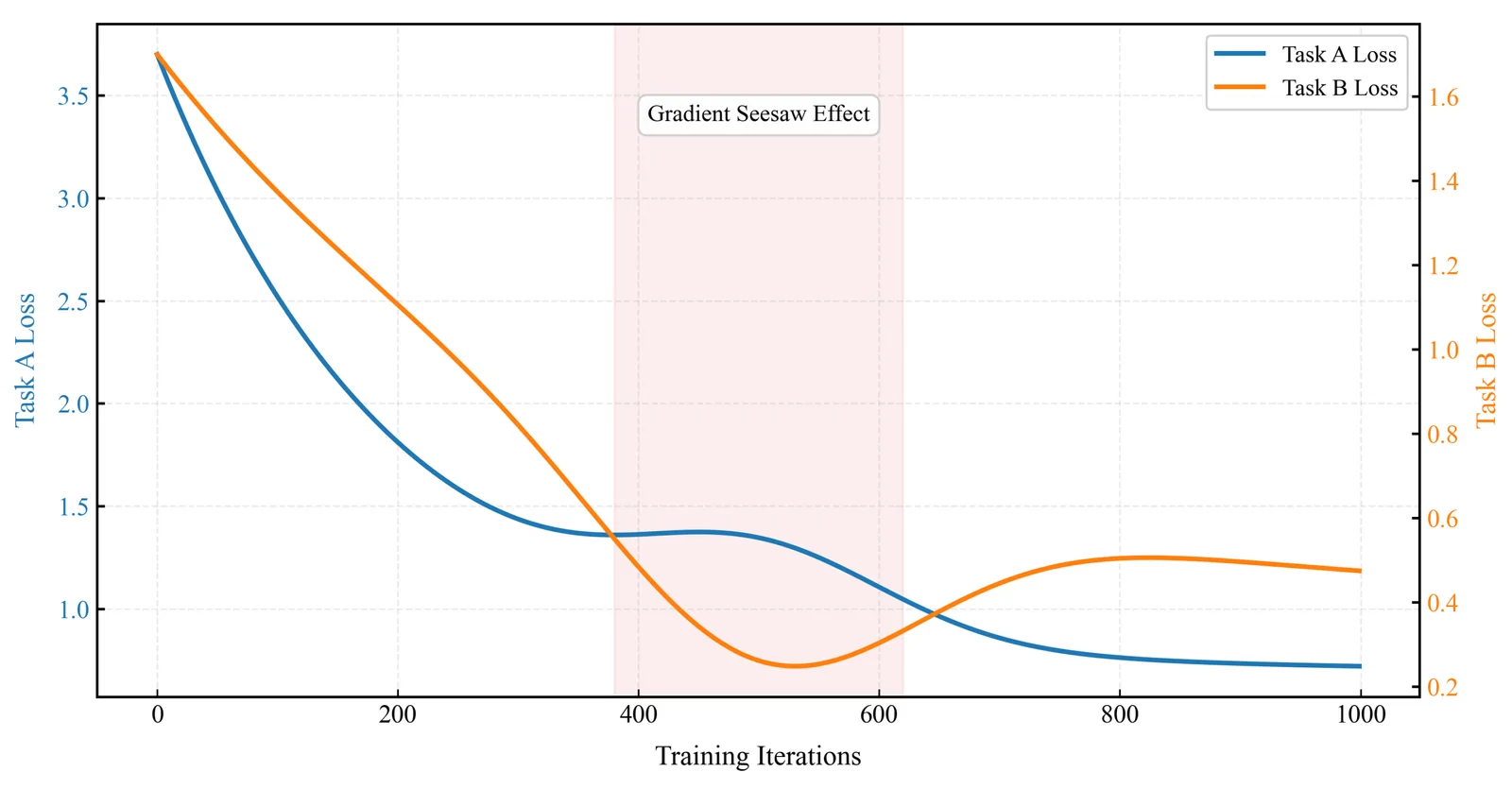
The gradient seesaw effect in multi-task joint optimization.

During the end-to-end training process of MTC-DETR, the network needs to simultaneously optimize two heterogeneous tasks: object detection and keypoint detection. To address the gradient seesaw effect (as illustrated in Figure 6) caused by inconsistent loss and convergence rates across tasks, this paper proposes the Homoscedastic Uncertainty Dynamic Joint Loss (HUDJ-Loss). It models the observation noise of each task as an independently learnable parameter, and adaptively weights the loss of each task based on dynamic uncertainty to coordinate optimization gradients across different branches, thereby ensuring stable global convergence.

#### 3.4.1. Object Detection Loss

To achieve end-to-end post-processing-free detection, the object detection loss $L_{det}$ is built upon bipartite graph global optimal matching based on the Hungarian algorithm. Assuming the prediction is $\hat{Y}={\left\{{\hat{y}}_{i}\right\}}_{i=1}^{N}$, and the ground truth is padded to the same size as $Y={\left\{y_{i}\right\}}_{i=1}^{N}$, the network first computes the pairwise matching cost consisting of classification error, bounding box L1 distance and Generalized Intersection over Union (GIoU) distance between each pair of prediction and ground truth. After solving for the optimal permutation $\hat{\sigma }$ that minimizes the total matching cost, the object detection loss is defined as the weighted sum of classification loss and bounding box regression loss:

(16) $$L_{det}=\sum_{i=1}^{N}\left[\lambda_{cls}L_{FL}\left(c_{i},{\hat{c}}_{\hat{\sigma }\left(i\right)}\right)+I_{\left\{c_{i}\neq \emptyset \right\}}\left(\lambda_{L1}{∥b_{i}-{\hat{b}}_{\hat{\sigma }\left(i\right)}∥}_{1}+\lambda_{giou}L_{giou}\left(b_{i},{\hat{b}}_{\hat{\sigma }\left(i\right)}\right)\right)\right]$$

where *N* is the total number of initialized object queries; $c_{i}$ and $b_{i}$ represent the ground truth class label and bounding box coordinates respectively; ${\hat{c}}_{\hat{\sigma }\left(i\right)}$ and ${\hat{b}}_{\hat{\sigma }\left(i\right)}$ denote the predicted class and bounding box matched under the optimal permutation $\hat{\sigma }$; $I_{\left\{c_{i}\neq \emptyset \right\}}$ is the indicator function, which outputs 1 for positive matched samples and 0 for background samples; $L_{FL}$ and $L_{giou}$ denote Focal Loss and GIoU Loss respectively; $\lambda_{cls}$, $\lambda_{L1}$ and $\lambda_{giou}$ are the corresponding predefined weight hyperparameters.

#### 3.4.2. Keypoint Detection Loss

In the graph topology-guided keypoint detection branch, the keypoint detection loss $L_{kpt}$ is designed as a weighted fusion of coordinate regression loss and Object Keypoint Similarity (OKS) loss. For the *j*-th keypoint, the loss is formulated as

(17) $$L_{kpt}=\sum_{j=1}^{K}I_{\left\{v_{j}>0\right\}}\left[\gamma_{L1}{∥p_{j}-{\hat{p}}_{j}∥}_{1}+\gamma_{OKS}\left(1-\exp\left(-\frac{∥p_{j}-{\hat{p}}_{j}{∥}_{2}^{2}}{2s^{2}\kappa_{j}^{2}}\right)\right)\right]$$

where *K* is the total number of defined human keypoints (K=17 under the COCO standard); $p_{j}$ and ${\hat{p}}_{j}$ represent the ground truth and predicted values of the *j*-th keypoint respectively; $I_{\left\{v_{j}>0\right\}}$ is the visibility mask; *s* denotes the area scale factor of the matched human bounding box from the detection branch; $\kappa_{j}$ is a per-keypoint normalization constant that controls the attenuation variance; $\gamma_{L1}$ and $\gamma_{OKS}$ are weight coefficients balancing L1 distance and OKS similarity.

#### 3.4.3. Multi-Loss Joint Optimization

Static manual weight tuning not only requires extensive hyperparameter exploration but also easily falls into a zero-sum trade-off, where improving one task’s performance comes at the cost of degrading the other. To address this issue, HUDJ-Loss introduces a dynamic weighting framework based on Bayesian homoscedastic uncertainty. It is derived by maximizing the Gaussian log-likelihood of multi-task outputs under the homoscedastic noise assumption.

Specifically, the prediction error of each task is assumed to follow a zero-mean Gaussian distribution with homoscedastic observation noise, where the noise level reflects the difficulty and measurement scale of the task. By treating the noise variance of each task as an independent learnable parameter and minimizing the negative log-likelihood of the joint prediction, the total loss function is obtained. For tasks τ∈{det,kpt}, the joint loss function is formally defined as

(18) $$L_{total}=\frac{1}{2}\exp\left(-s_{det}\right)L_{det}+\frac{1}{2}\exp\left(-s_{kpt}\right)L_{kpt}+\frac{1}{2}\left(s_{det}+s_{kpt}\right)$$

where $L_{det}$ and $L_{kpt}$ are the task-specific losses defined in the preceding subsections; $s_{\tau }=\log\left(\sigma_{\tau }^{2}\right)$ represents the log-variance parameter of the homoscedastic observation noise $\sigma_{\tau }$ for task τ. The log-variance form is adopted instead of directly using the standard deviation $\sigma_{\tau }$ to ensure numerical stability during backpropagation and avoid division-by-zero risks. The two log-variance parameters are updated end-to-end along with all other network parameters during training. The $\exp(-s_{\tau })$ term acts as an inverse-variance adaptive weight for each task, automatically adjusting the contribution of each task to the total loss according to its current convergence state. Meanwhile, the additive term $\frac{1}{2}\left(s_{det}+s_{kpt}\right)$ serves as a structural risk penalty, which constrains the network from reducing the total loss by infinitely amplifying the task uncertainty.

Compared with the classical general homoscedastic uncertainty framework, the proposed HUDJ-Loss makes targeted adaptation for the heterogeneous dual-task fall detection scenario. The classical framework typically assigns an independent uncertainty parameter to each individual loss term. In this work, the object detection task involves multiple sub-losses, including classification, bounding box L1 regression and GIoU Loss, and the keypoint detection task also contains coordinate regression and OKS loss. To avoid parameter redundancy and weight disorder among multiple sub-losses, one unified uncertainty parameter is allocated for each major task. This design achieves better compatibility with the parallel dual-branch architecture, and maintains stable gradient coordination between the two heterogeneous tasks.

## 4. Results

### 4.1. Experimental Settings

#### 4.1.1. Datasets and Splitting Strategies

In terms of dataset selection, this work focuses on vision-based fall detection. Multi-modal datasets such as UP-Fall [37] and UR-Fall [38] are not included, which rely on wearable sensors or environmental sensing devices. Accordingly, three pure visual datasets are adopted under a cross-domain evaluation protocol:

- CAUCAFall Dataset [16]: The CAUCAFall dataset contains various simulated fall actions and highly confusing activities performed by 10 volunteers of different genders, ages, heights and body mass indexes (BMIs). To eliminate subject appearance bias, CAUCAFall adopts a cross-subject evaluation protocol, focusing on verifying the model’s generalization ability to unseen individuals, preventing the model from overfitting to the appearance features of specific subjects. Images from 60% of volunteers are assigned to the training set, 10% are used for validation, and the remaining 30% form the test set.
- DiverseFall10500 Dataset [17]: This is a large-scale and diverse benchmark dataset specifically constructed for intelligent fall detection research. It consists of 10,500 annotated samples covering various complex indoor scenarios, including wards, corridors, living rooms, and stairwells, with diverse lighting conditions, shooting angles, and background layouts. Adopting a cross-scene evaluation protocol, the dataset aims to validate the environmental adaptability and robustness of fall detection models in real-world scenarios. All samples are annotated to distinguish fall and non-fall behaviors, and the dataset is divided into training, validation, and test sets at a scenario-based ratio of 7:1:2.
- MT-Fall Dataset: The custom MT-Fall dataset is constructed to evaluate model performance on long-tail samples with multi-person mutual occlusion and variable surveillance depth of field. Collected from public image repositories, it includes 7782 images covering real-world scenarios such as streets, indoor spaces and sports fields. The dataset contains 22,314 annotated human instances in total, among which 2911 are fall samples and 19,403 are normal samples. During annotation, a pre-trained YOLO26 model was used for initial bounding box generation, followed by manual correction and supplementary annotation.The dataset is also split into training, validation and test sets at a 7:1:2 ratio. MT-Fall dataset focuses on the evaluation of complex real scenes, with dense multi-person occlusion, wide depth-of-field scale differences and long-tailed class distribution. Detailed statistics of the dataset are summarized in Table 1, where three occlusion levels are defined based on the ratio of visible keypoints among 17 standard COCO-format human keypoints: mild occlusion refers to a visible keypoint ratio above 80% (at most 3 keypoints occluded), moderate occlusion corresponds to a ratio of 50%–80% (4–8 keypoints occluded), and severe occlusion means a ratio below 50% (more than 8 keypoints occluded).

Since none of the datasets natively contained keypoint annotations, the state-of-the-art OpenPose was utilized to perform automated keypoint annotation across all datasets with manual rectification. The generated keypoint annotations conform to the standard COCO format, featuring 17 human body keypoints. During the data preprocessing stage, in addition to conventional random scaling and color jitter, Mosaic and MixUp augmentations are introduced to enhance the diversity of backgrounds in the images.

#### 4.1.2. Evaluation Metrics

The evaluation metrics are categorized into three tasks: object detection, keypoint detection, and fall state classification:

Object Detection Metrics: For human bounding box detection, the mean Average Precision at Intersection over Union (IoU) thresholds of 0.5 and 0.5:0.95 (denoted as mAP@0.5 and mAP@0.5:0.95, respectively) are adopted as the primary evaluation metrics.

Keypoint Detection Metric: Object Keypoint Similarity (OKS) is employed as the keypoint detection metric, which is computed as

(19) $$OKS=\frac{\sum_{i}\exp\left(-d_{i}^{2}/2s^{2}\kappa_{i}^{2}\right)\delta \left(v_{i}>0\right)}{\sum_{i}\delta \left(v_{i}>0\right)}$$

where $d_{i}$ is the Euclidean distance between the ground truth and the predicted keypoint, *s* is the scale factor of the human target, $\kappa_{i}$ is the normalization constant for different types of keypoints, and $v_{i}$ is the visibility flag of the keypoint.

Fall Classification Metrics: The binary action state (fall vs. normal) is evaluated using precision (*P*) and recall (*R*). This classification result is derived from the object detection task. Specifically, an image is classified as a “Fall” event if at least one human within it is recognized as falling. The mathematical definitions are as follows:

(20) $$P=\frac{TP}{TP+FP},\;R=\frac{TP}{TP+FN}$$

where TP (True Positive) is the number of correctly detected fall events, FP (False Positive) represents false alarms (normal events misclassified as falls), and FN (False Negative) represents missed fall events.

#### 4.1.3. Implementation Details

Hardware and Software Environment: All experiments are conducted on a graphics workstation equipped with a single NVIDIA RTX 4090 GPU (24GB VRAM) and an Intel Core i9-13900K CPU. The operating system is Ubuntu 22.04 LTS, and the deep learning framework is PyTorch 2.0.1 paired with CUDA 11.8.

Training Hyperparameters: The backbone of MTC-DETR is initialized with RepViT-M2.3 pre-trained on ImageNet. The network is trained using the AdamW optimizer, with an initial learning rate set to $1\times 10^{-4}$ and a weight decay coefficient of $1\times 10^{-4}$. The model is trained for a total of 150 epochs. To prevent gradient explosion during the initial training phase, a linear warmup strategy is applied for the first three epochs, followed by a cosine annealing algorithm to smoothly decay the learning rate to $1\times 10^{-6}$. The input resolution is 640×640 and the batch size is set to 4.

To ensure fair and reliable comparisons, all baseline methods adopt exactly the same training protocol as described above. All models are implemented and evaluated under the same hardware and software environment to rule out performance discrepancies caused by implementation conditions.

### 4.2. Visual Characteristics Analysis of Falls

**Figure 7:**
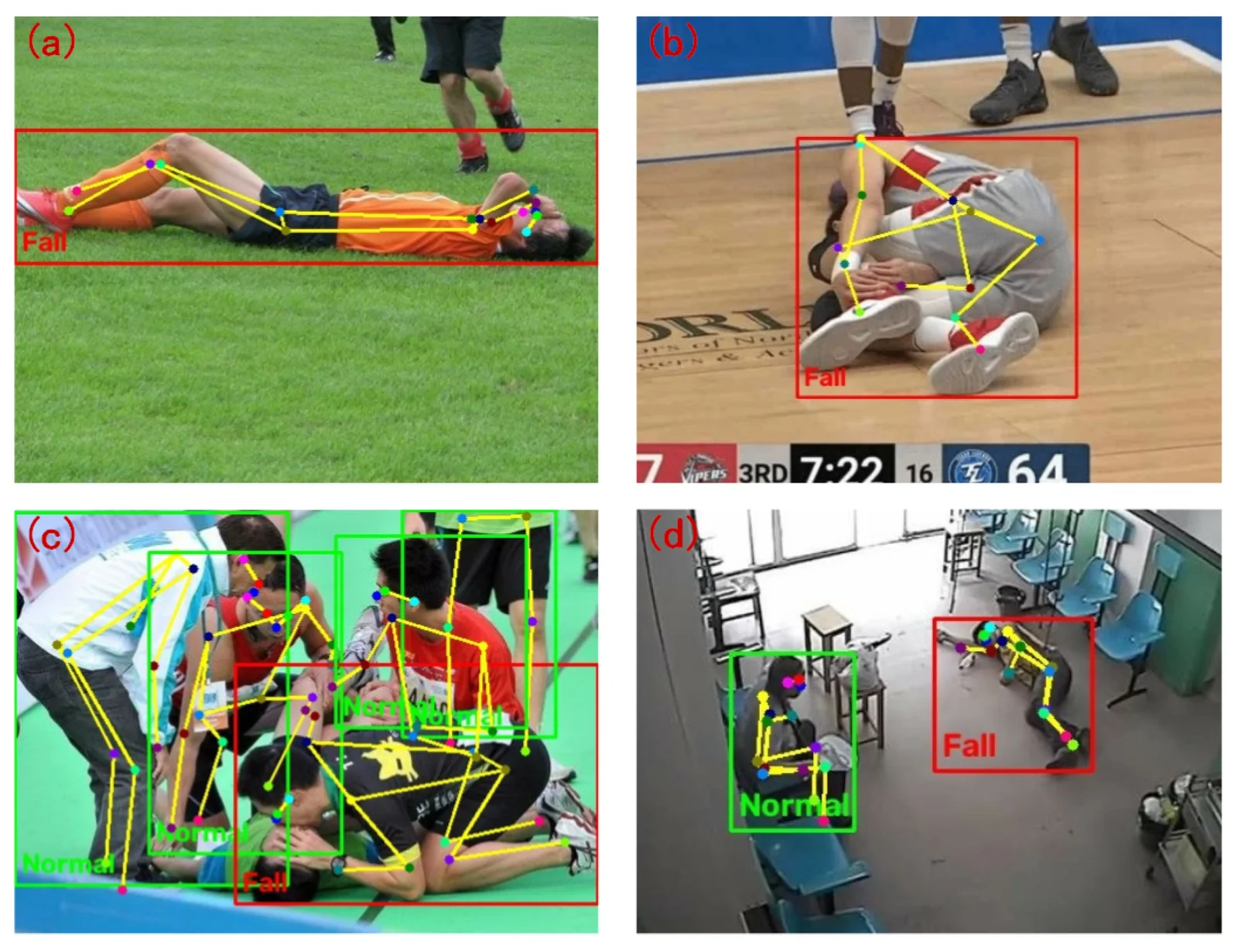
Visual characteristics of falls under different scenarios. (**a**) Typical fall from unoccluded front view; (**b**) crouched fall posture; (**c**) fall in multi-person occlusion scene; (**d**) fall from long-distance top-down monitoring perspective.

As shown in Figure 7, four groups of fall samples in different scenarios present the visual characteristics of fall events from multiple dimensions. The bounding boxes and keypoints are generated by YOLO-Pose [39]. Figure 7a shows a typical fall posture from an unoccluded front view, which is the most complete presentation of fall characteristics: the human body stretches horizontally as a whole, the bounding box aspect ratio increases significantly, the body center of gravity drops close to the ground, and core keypoints such as shoulders, hips, knees and ankles present an approximately horizontal extended distribution. The appearance contour and keypoint distribution together constitute the most recognizable visual features of falls. Figure 7b shows a crouched fall posture: due to protective movements or collision, the limbs are flexed and gathered, the bounding box aspect ratio is significantly reduced, and the overall spatial distribution of keypoints is compressed, which is similar to postures such as squatting and kneeling in appearance. Figure 7c illustrates a fall incident surrounded by a crowd of rescuers. The actual fallen person on the ground is severely occluded by surrounding people, with their full body contour and complete keypoint distribution almost invisible, resulting in easily missed detection. Meanwhile, the kneeling rescuer in the foreground exhibits a bounding box morphology and keypoint spatial distribution similar to those of a falling posture, which tends to be misclassified as a fall target. Figure 7d illustrates a fall from a long-distance top-down monitoring perspective, where fall features show perspective deformation: affected by the imaging projection effect, the overall distribution of human keypoints presents an approximately vertical morphology, and the bounding box aspect ratio feature is compressed and weakened.

Across these varied real-world scenarios, fall events exhibit distinct visual patterns. Characteristics such as bounding box aspect ratio, body center-of-gravity height and keypoint spatial layout are straightforward to observe in clear front-view scenes, and are the basis for conventional fall detection. However, these appearance-based characteristics degrade noticeably when facing occlusion, perspective distortion or crouched postures, and may even lead to missed detection or false alarms in complex scenes. In comparison, the inherent connectivity and kinematic constraints between human keypoints maintain relatively stable structural properties regardless of partial occlusion, viewing angle or posture deformation. These structural features can still provide valid reference for posture inference even when local appearance information is incomplete.

### 4.3. Comparative Experiments

To validate the effectiveness of MTC-DETR, the compared models are categorized into three groups—object detection methods, keypoint detection methods, and multi-task learning methods:

- Object Detection Methods: This category includes Transformer-based detectors, including DETR [4], DINO-DETR [40], RT-DETR [5], and RT-DETRv4 [41], alongside CNN-based real-time detectors YOLO11 [42] and YOLO26 [43].
- Keypoint Detection Methods: ViTPose [44], RTMPose [45], and ED-Pose [46] are chosen as keypoint detection models. Since these models are designed for keypoint output, a lightweight Multi-Layer Perceptron (MLP) classification head is appended to their terminal feature maps to predict fall probabilities.
- Multi-Task Learning Methods: To evaluate the proposed method against existing collaborative optimization paradigms, the hard parameter sharing framework HPS-MTL [47] and the end-to-end YOLO-Pose [39] are selected. Furthermore, recent multi-task optimization strategies are introduced for comparison, including scMoE [48], MoDo [49], and Nash-MTL [50].

#### 4.3.1. CAUCAFall Dataset

The CAUCAFall dataset employs a cross-subject splitting strategy, aiming to evaluate the model’s generalization capability to unseen subjects. As shown in Table 2, mainstream object detectors represented by RT-DETRv4 achieve competitive baseline performance with strong backbone feature extraction. However, these methods rely heavily on pixel-level appearance features. When facing unseen individuals, this leads to a drop in recall, as fall actions are often misclassified as daily activities.

The proposed MTC-DETR achieves the best results on all detection and keypoint metrics, with 92.7% mAP@0.5 and 91.8% OKS. Compared with the baseline RT-DETR, it improves mAP@0.5 by 5.2%, and also outperforms the multi-task method Nash-MTL by 1.5%. For keypoint detection, the TG-LGSA models human skeleton connectivity via graph convolutions, which helps the model to recognize fall patterns based on kinematic structure rather than superficial appearance features. For object detection, the pose prior passed from the keypoint branch provides additional geometric constraints for the object detection branch, further improving localization robustness. Notably, both tasks obtain consistent performance gains without obvious trade-off. This is because the encoder outputs task-specific adapted features for both branches. Combined with the HUDJ-Loss that stabilizes multi-task training convergence, the framework reduces the interference of individual differences, and achieves better cross-subject generalization.

#### 4.3.2. DiverseFall10500 Dataset

Different from the cross-subject protocol that focuses on individual appearance differences, the DiverseFall10500 benchmark primarily examines the model’s resilience to environmental changes.

As shown in Table 3, all baseline methods suffer from performance degradation. For single-task object detectors, the difference of shooting distances causes scale differences among human instances, while the traditional multi-scale fusion structure cannot dynamically adjust receptive fields. For keypoint-based methods, viewpoint changes distort the two-dimensional projection distribution of human keypoints, breaking the spatial posture patterns learned from training views. The proposed MTC-DETR achieves the best performance on all evaluation metrics, with 88.9% mAP@0.5 and 89.5% OKS. Compared with the baseline RT-DETR, it achieves a 5.7% improvement in mAP@0.5, and its performance decay from cross-subject to cross-scene settings is smaller than most baselines. The performance gain comes from two perspectives. The graph topology prior maintains the effectiveness of posture reasoning under viewpoint deflection, and the cross-branch pose prior further corrects bounding box localization drift caused by perspective distortion.

#### 4.3.3. MT-Fall Custom Dataset

The MT-Fall custom dataset is characterized by dense multi-person crowds, inter-person occlusion, wide depth-of-field variations and a long-tailed class distribution. As shown in Table 4, single-task object detectors suffer from obvious recall degradation. When the landmarks such as the heads are obscured, these appearance-driven models lose the discriminative features to distinguish falls from normal postures. Since keypoint-based methods rely on visible pixel evidence to locate keypoints, heavily occluded body parts produce inaccurate keypoint outputs, which in turn weaken downstream fall classification performance. In addition, most existing multi-task frameworks lack explicit structural complementation. Even gradient balancing strategies can only optimize loss weighting, but cannot compensate for the fundamental feature loss caused by occlusion.

**Figure 8:**
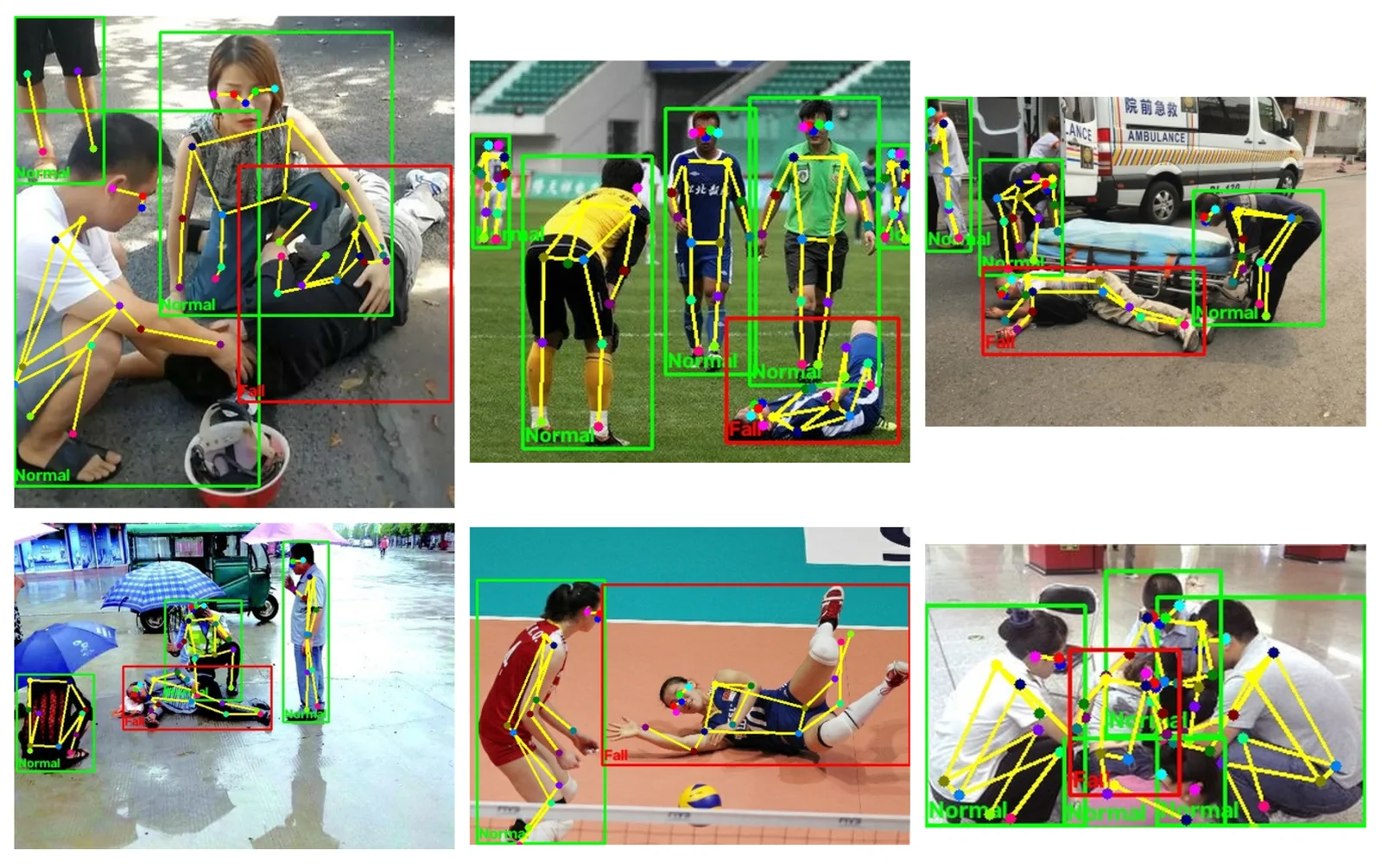
Result visualization of MTC-DETR on the MT-Fall Dataset.

MTC-DETR achieves state-of-the-art performance across all metrics, with 90.4% mAP@0.5 and 89.3% OKS. It outperforms the baseline RT-DETR by 11.0% in mAP@0.5, and surpasses the multi-task baseline Nash-MTL by 4.6%. The performance gain demonstrates that the topology prior can compensate for the visual feature loss caused by inter-person mutual occlusion. The visualization results in Figure 8 further verify the robustness of MTC-DETR in complex real scenarios. In crowded scenes with severe inter-person occlusion, the model can accurately identify fall postures even when limb contours overlap heavily. Benefiting from the topology-guided reasoning, the keypoint branch can still infer the positions of occluded keypoints, which in turn assists the object detection branch. In addition, it can simultaneously detect both large human targets and small distant targets, with no missed detection of small-scale fall events, verifying the effectiveness of the dynamic scale-routing encoder for cross-scale human targets. Particularly noteworthy is the 90.6% recall rate of MTC-DETR, which leads all compared methods. From the perspective of practical deployment, recall directly characterizes the model’s capture capability for real fall events, which fits the core demand of risk control in fall monitoring scenarios. In application scenarios such as elderly care institutions and public video surveillance, missed fall detection may lead to delayed rescue and even irreversible safety consequences. For this reason, maintaining a high recall rate to reduce missed alarms has more practical value than simply optimizing overall detection accuracy. In practical elderly care and public surveillance scenarios, missed fall alarms carry higher safety risks than false alarms. The MTC-DETR reduces the missed detection in crowded and complex environments, proving practical deployment value compared with methods that only optimize for overall accuracy.

### 4.4. Inference Efficiency and Complexity Analysis

In this section, further comparison experiments are conducted on the MT-Fall dataset to evaluate the inference efficiency and complexity of different methods. All methods are evaluated with parameter count (Params), floating-point operations (FLOPs) and inference frames per second (FPS). The results are summarized in Table 5.

**Figure 9:**
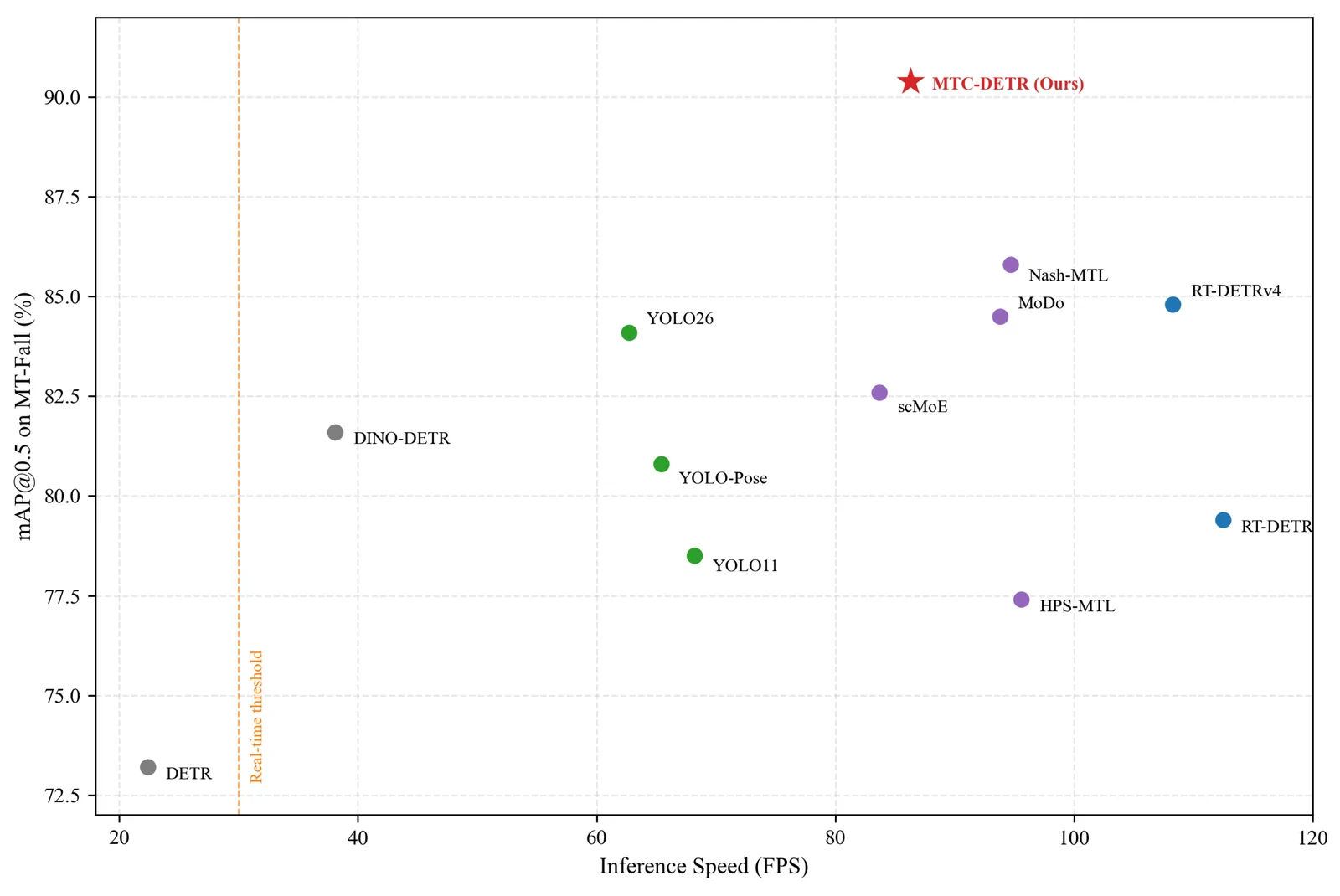
Accuracy–efficiency trade-off on the MT-Fall dataset.

MTC-DETR contains 29.5M parameters and 41.3G FLOPs, showing a moderate increase compared with the baseline RT-DETR, which is mainly attributed to the dual-branch decoder structure and the topology modeling modules. Most methods satisfy the basic real-time requirement of 30 FPS. Among multi-task learning approaches, MTC-DETR achieves higher inference speed than YOLO-Pose and scMoE, and exhibits comparable speed with other multi-task baselines that only introduce gradient optimization strategies. It achieves the highest detection accuracy with controllable computational overhead, and reaches a competitive accuracy–efficiency trade-off. The corresponding trade-off relationship is visualized in Figure 9.

### 4.5. Ablation Studies

To verify the efficacy of each module and discuss the hyperparameters, this section conducts ablation studies on the MT-Fall dataset. The baseline model is constructed based on the baseline RT-DETR with a plain parallel dual-branch decoder that performs object detection and keypoint detection independently, and a fixed-weight joint loss function.

#### 4.5.1. Effectiveness of Core Components

Starting from the baseline, five modules are added progressively. Training hyperparameters, data augmentation strategies and input resolution remain consistent across all experiments.

As shown in Table 6, both detection and keypoint detection performance improve steadily with the addition of each module, verifying the necessity of all proposed designs:

- DLKA improves multi-scale human feature representation via adaptive long-range enhancement, improving mAP@0.5 by 2.7% over the baseline.
- Further integration of DCF-TAD enables the encoder to perform dynamic multi-scale feature fusion and task-specific feature calibration, delivering an additional 2.6% gain in mAP@0.5.
- The TG-LGSA module enhances the keypoint branch with occluded keypoint reasoning capability via graph topology modeling, pushing OKS to 83.5%.
- The cross-branch pose prior injection mechanism injects skeletal topology features from the keypoint branch into the object detection branch, guiding the sampling trajectory of Deformable Cross-Attention in the detection branch. With all other modules kept unchanged, this step brings a 2.6% improvement in mAP@0.5 for object detection. And the OKS also increases by 3.7%, which quantitatively verifies the enhancement effect of pose information on detection accuracy.
- Finally, HUDJ-Loss alleviates multi-task gradient conflicts and supports the synchronous optimization of both tasks. The complete MTC-DETR achieves 90.4% mAP@0.5 and 89.3% OKS, indicating that dynamic loss balancing improves the performance potential of multi-task collaboration.

#### 4.5.2. Ablation Study on the DLKA Module

To quantitatively verify the contribution of each core design in DLKA, ablation experiments are conducted on the multi-task RT-DETR baseline. To ensure controlled variables, the vanilla parallel dual-branch decoder and fixed-weight joint loss remain unchanged across all groups, and only the feature enhancement module within the encoder is replaced. There are in total four designs for evaluation: the decomposed large-kernel structure, cross-scale feature projection, deformable sampling mechanism, and gated fusion mechanism. The detailed settings are as follows:

- Baseline (AIFI): Original RT-DETR encoder with the Attention-Based Intra-Scale Feature Interaction (AIFI) module only applied on the highest-level feature map $C_{5}$, serving as the performance baseline.
- LKA: AIFI is replaced with standard decomposed large-kernel attention (LKA), which is deployed on all three scales $\left\{C_{3},C_{4},C_{5}\right\}$.
- LKA + CSP: Cross-Scale Projection (CSP) is added on the LKA to enable information interaction across different feature levels and is only performed on $C_{5}$.
- LKA + CSP + DS: Deformable Sampling (DS) is introduced into depthwise convolutions based on LKA + CSP, replacing fixed convolution kernels with deformable ones (as is presented in Figure 3).
- Full DLKA: Further adds learnable gated fusion (GF) for the cross-scale projection on $C_{5}$ based on the previous configuration, forming the complete DLKA module.

**Figure 10:**
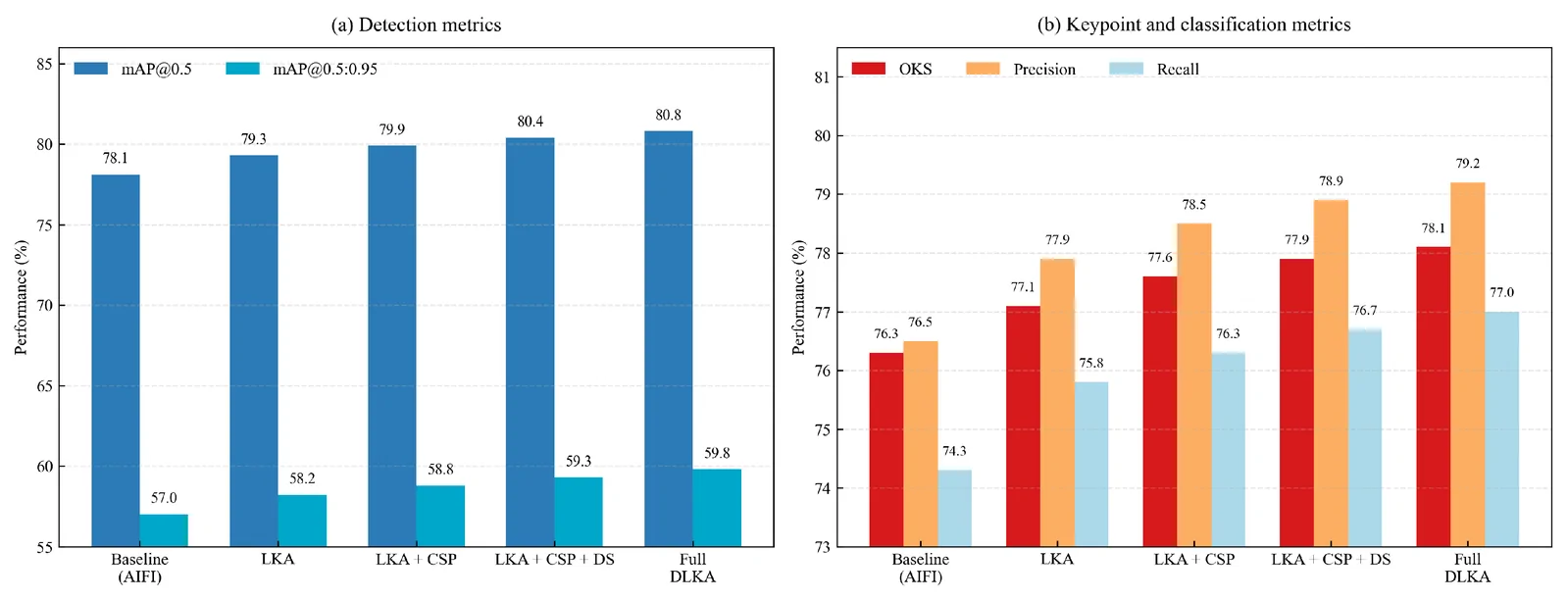
Ablation results of the DLKA module on the MT-Fall dataset.

As shown in Figure 10, standard LKA improves mAP@0.5 from 78.1% to 79.3% and mAP@0.5:0.95 from 57.0% to 58.2%. This indicates that the large-kernel attention can also expand the receptive field and enhance the ability for global modeling. After adding cross-scale projection (LKA + CSP), the two detection metrics only further increase by 0.6%, with a relatively limited gain. The reason lies in the differences in semantic granularity and response intensity between features at different levels. With the introduction of deformable sampling (LKA + CSP + DS), mAP@0.5 and mAP@0.5:0.95 are further increased. The improvement demonstrates that learnable sampling offsets can adaptively align with the geometric contours. The full DLKA with learnable gated fusion finally achieves 80.8% and 59.8% on the two detection metrics, along with an OKS of 78.1%. The gating mechanism suppresses interference from cross-scale features in cross-scale projection through weight constraints, ensuring the dominance of native-scale features.

#### 4.5.3. Ablation Study on the DCF-TAD Module

A 3 × 3 ablation experiment is conducted on the baseline with the DLKA module and CCFM. Two independent dimensions are set as variables: cross-scale fusion architecture and task-aware scheduling strategy. The former includes three configurations: original static cascaded CCFM (CCFM), static parallel dual-path fusion (SP-DP, DCF without dynamic routing), and dynamic cross-scale fusion (DCF). The latter includes no scheduling with shared features (NS), squeeze-and-excitation-based calibration (SE), and task-aware dispatch (TAD), resulting in a total of nine controlled configurations.

**Figure 11:**
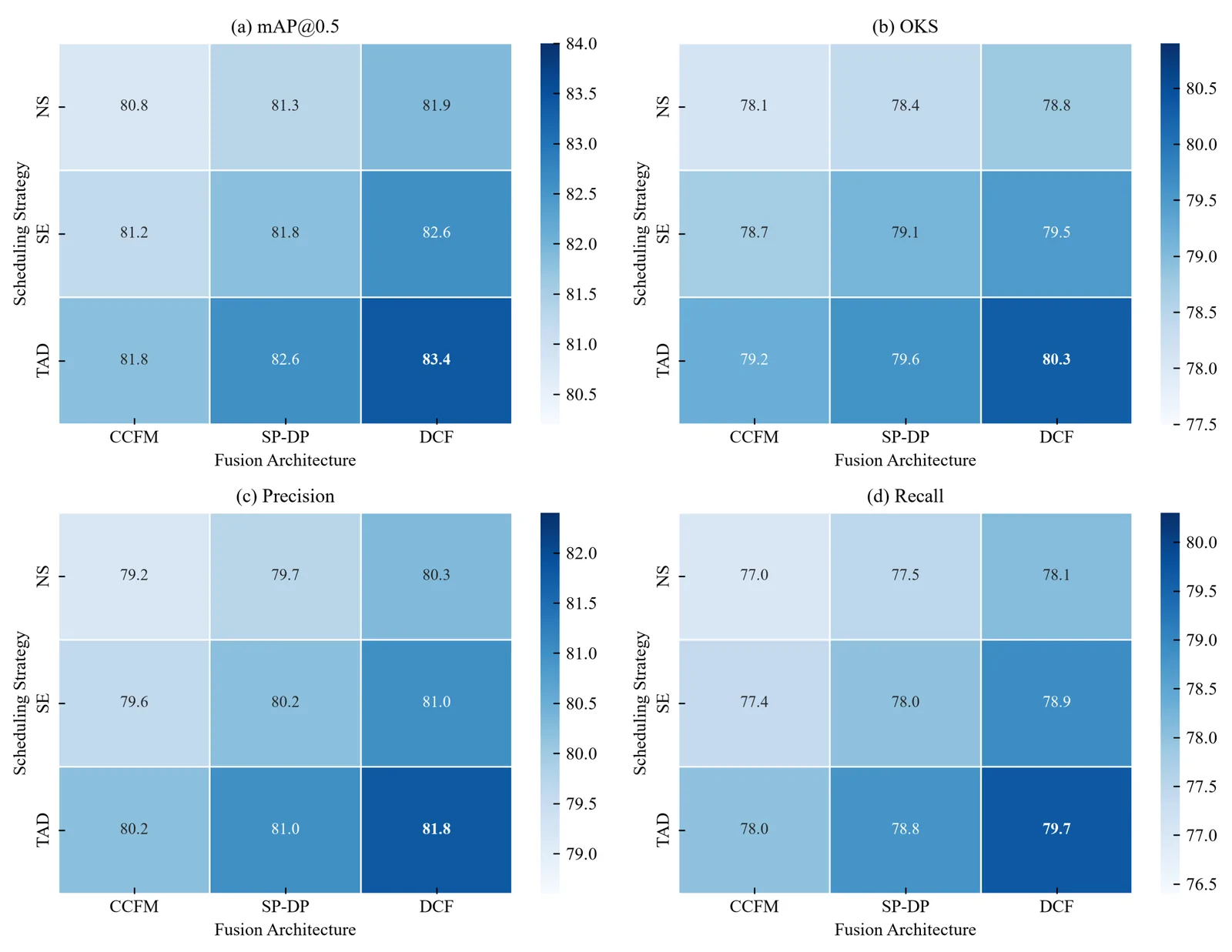
Factorial ablation results of the DCF-TAD module on the MT-Fall dataset. (**a**) mAP@0.5; (**b**) OKS; (**c**) precision; (**d**) recall.

As shown in Figure 11, performance on all metrics improves consistently as the fusion architecture upgrades from CCFM to DCF, with larger gains observed on mAP@0.5 than on OKS. Under a fixed fusion architecture, upgrading the task-aware scheduling strategy also improves performance. When dynamic cross-scale fusion is combined with task-aware dispatch, the total improvement in mAP@0.5 reaches 2.6%. High-quality multi-scale features provides the foundation for fine-grained task scheduling, and differentiated feature distribution further improves the multi-task architecture.

#### 4.5.4. Ablation Study on the TG-LGSA Module

In the TG-LGSA ablation experiments, the baseline is a keypoint decoder with vanilla self-attention without graph topology modeling. As shown in Table 7, topology priors primarily improve keypoint localization accuracy. Adding the multi-hop GCN improves OKS by 2.6% and mAP@0.5 by 1.1%, verifying the effectiveness of human kinematic topology constraints for occluded pose reasoning. On this basis, topological positional encoding (TPE) derived from the graph Laplacian is injected into keypoint queries, which reduces the matching ambiguity of occluded keypoints and yields a 0.3% improvement in OKS. Subsequently, global spatial attention with shortest-path distance (SPD) penalty is introduced to supplement global pose consistency modeling and suppress invalid cross-keypoint attention associations, bringing an additional 0.4% OKS gain. The adaptive gated fusion mechanism performs the dynamic weighting of local topological features and global spatial features, reducing the adaptation bias caused by fixed fusion weights. The complete TG-LGSA module achieves 83.5% OKS and 85.0% mAP@0.5.

Parameter sensitivity analysis is further performed on three core hyperparameters: multi-hop count *S*, adjacency balance factor α, and distance penalty coefficient γ.

**Figure 12:**
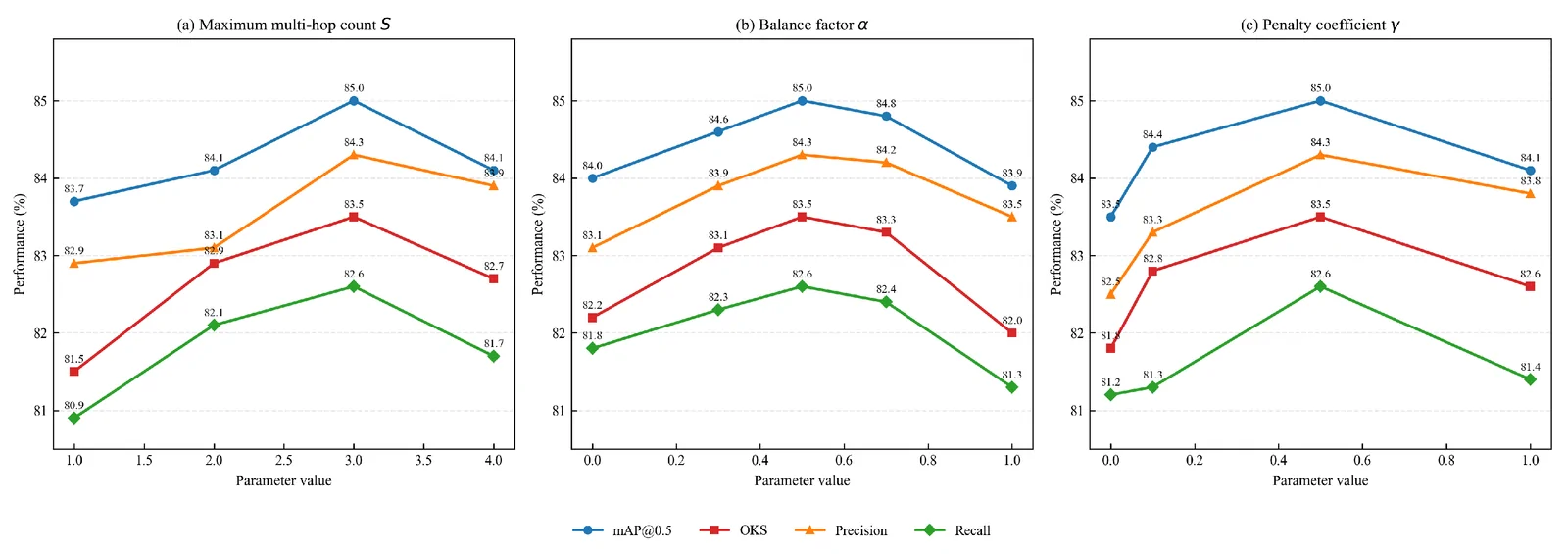
Sensitivity analysis of key parameters in TG-LGSA on the MT-Fall dataset. (**a**) Maximum multi-hop count *S*; (**b**) balance factor α; (**c**) penalty coefficient γ.

As shown in Figure 12, the multi-hop count achieves performance peaks at S=3. For the multi-hop count *S*, the human skeleton forms a hierarchical topology centered on the trunk. The shortest path from core trunk keypoints to distal limb keypoints (e.g., wrist, ankle) typically spans 2 to 3 hops. Theoretically, setting S=3 enables multi-hop graph convolution to fully cover the entire skeletal connection chain from trunk to extremities, which allows the model to infer occluded distal keypoints using features of adjacent visible keypoints. A smaller *S* fails to cover long-range skeletal connections and limits occlusion reasoning capability, while an excessively large *S* leads to over-smoothing of features and loses discriminative information of individual keypoints.

For the adjacency balance factor α, fall postures exhibit dual attributes: they are constrained by inherent human kinematic connections on the one hand, and involve diverse non-rigid deformations on the other. Accordingly, the model needs to balance static physical skeleton priors and dynamic semantic correlations to adapt to various falling poses. Theoretically, a moderate weight for both components is required. Experimental results verify that the optimal value α=0.5 achieves the best trade-off, matching the dual characteristics of fall movements.

For the distance penalty coefficient γ, the 17 human keypoints have a moderate node density. The shortest-path distance penalty is designed to suppress invalid attention between physically unrelated keypoints and reduce cross-person misassociation in crowded scenes. Theoretically, an excessively large penalty would restrict the model from capturing global posture consistency, while an excessively small penalty would fail to suppress noise interference. The optimal value γ=0.5 strikes a proper balance: it retains the long-range modeling ability of global attention while effectively suppressing invalid associations, adapting well to the spatial distribution of human skeletal keypoints.

#### 4.5.5. Ablation Study on the HUDJ-Loss

This section aims to verify the effectiveness of HUDJ-Loss in alleviating gradient conflicts and balancing heterogeneous multi-task optimization. Four representative multi-task loss balancing methods are selected as baselines for horizontal comparison:

- Equal Weight: Detection loss and keypoint loss are directly summed with a fixed 1:1 ratio, serving as the basic multi-task loss configuration.
- Fixed Weight: Optimal constant weight coefficients obtained through grid search.
- GradNorm: GradNorm is a classic dynamic weighting method that balances the optimization pace of different tasks by aligning their gradient norms.
- DWA (Dynamic Weight Average): DWA is a dynamic weighting strategy that adjusts task weights according to the descending rate of each task loss.

Experimental results are summarized in Table 8. The equal weight scheme achieves the worst overall performance, as the keypoint branch dominates backpropagation, resulting in insufficient optimization of the detection task, as discussed in Section 4.6. Manually tuned fixed weights achieve a notable improvement, but require extensive hyperparameter exploration and cannot adapt to the dynamic optimization states at different training stages. Both GradNorm and DWA deliver further gains via dynamic weighting, but their adjustment mechanisms based on gradient magnitude or loss change rate lack an explicit modeling of task-specific observation noise, yielding limited improvement on heterogeneous detection-and-keypoint tasks.

The proposed HUDJ-Loss achieves the best performance, with mAP@0.5 reaching 90.4% and OKS reaching 89.3%. Compared with the fixed-weight baseline, it improves mAP@0.5 by 2.8% and OKS by 2.1%, and both tasks obtain synchronous performance gains without the “gradient seesaw” effect, where one task improves at the cost of the other. This confirms that, by modeling task-specific homoscedastic uncertainty as learnable parameters, HUDJ-Loss can adaptively suppress high-noise task gradients in the early training stage and strengthen the fine-grained optimization of converged tasks in the later stage.

#### 4.5.6. Performance Evaluation Across Occlusion Severity Levels

To quantify model performance under different occlusion conditions, evaluation is conducted on the MT-Fall test set based on existing keypoint visibility annotations. Three occlusion severity levels are defined according to the visible keypoint ratio of each human instance, with detailed statistics provided in Table 1.

For images containing multiple human instances with varying occlusion degrees, each instance is assigned exclusively to the group corresponding to its own occlusion level. Evaluation metrics are calculated independently on each instance subset without splitting original image files. This grouping scheme aligns with the instance-level evaluation paradigm of object detection and keypoint detection tasks, and avoids statistical bias caused by assigning a uniform occlusion label to all instances in a single image. Three representative methods are selected as baselines: RT-DETR, YOLO-Pose, and Nash-MTL. Two core metrics, mAP@0.5 for object detection and OKS for keypoint detection, are reported for each group.

As shown in Table 9, the performance of all compared methods degrades as occlusion severity increases, with consistent trends across both mAP@0.5 and OKS metrics. MTC-DETR achieves the best performance across all three occlusion levels, and exhibits a smaller performance decline from mild to severe occlusion compared with baseline methods. Specifically, under severe occlusion, MTC-DETR outperforms Nash-MTL by 8.8% in mAP@0.5 and 6.9% in OKS. The results indicate that the graph topology prior mitigates keypoint localization degradation caused by occlusion, and the cross-branch pose prior injection mechanism further transfers the structural cues to the detection branch, achieving synchronous performance improvement for both tasks under occlusion.

### 4.6. Training Convergence Visualization

**Figure 13:**
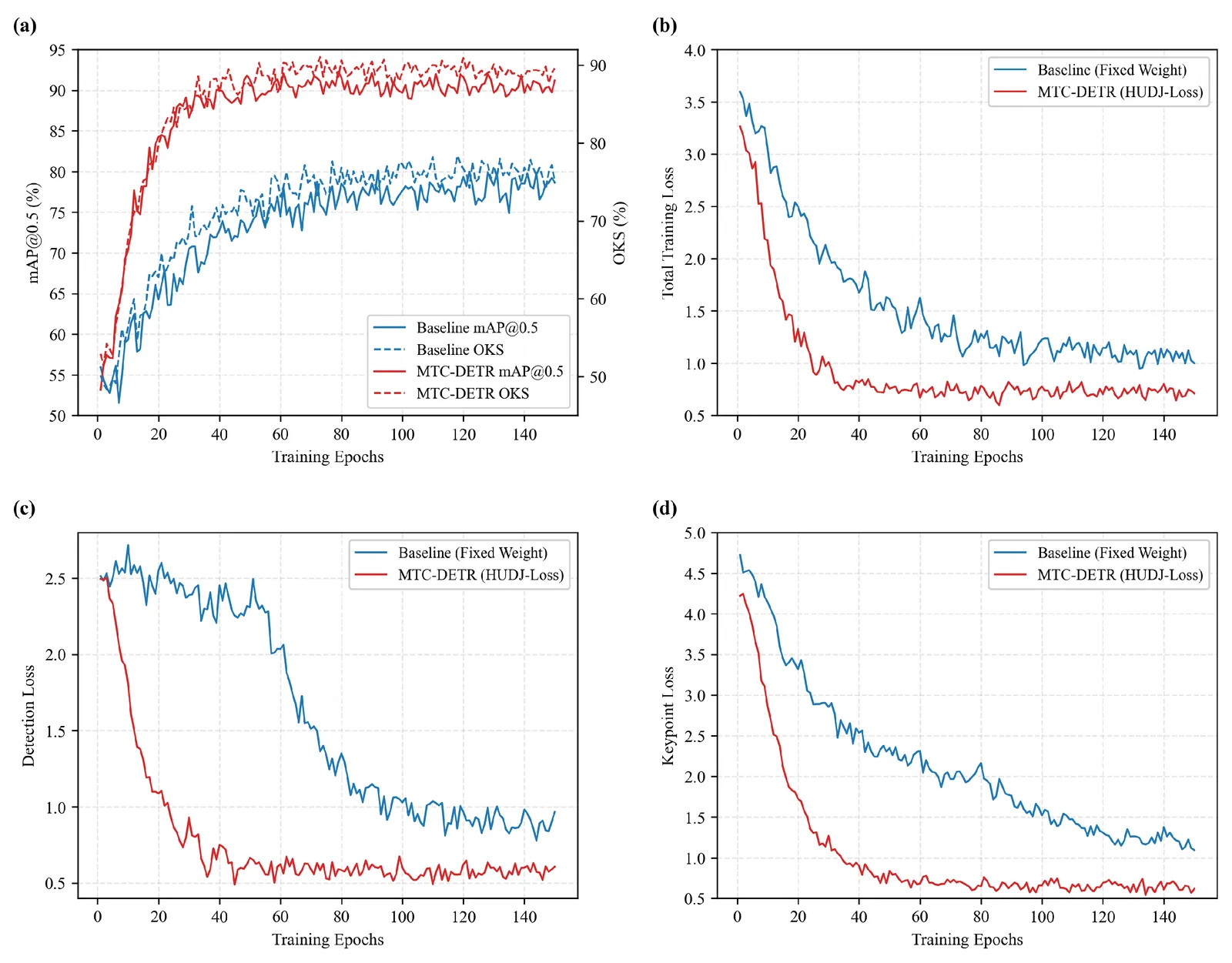
Training convergence curves on the MT-Fall dataset. (**a**) Detection mAP@0.5 and keypoint OKS curves; (**b**) total training loss curves; (**c**) object detection branch loss curves; (**d**) keypoint detection branch loss curves.

Training curves of RT-DETR and MTC-DETR are compared to illustrate the gradient balancing effect of the proposed HUDJ-Loss in Figure 13. As shown in Figure 13a, the baseline model exhibits fluctuations and slow convergence, with performance reaching a stable plateau around 70 epochs. In contrast, MTC-DETR converges to a stable performance interval at 30–40 epochs with smaller fluctuations, and achieves synchronous improvement on both detection and keypoint tasks. Figure 13b shows that the total loss of MTC-DETR decreases faster and more smoothly.

Figure 13c,d display per-task loss curves, presenting the gradient seesaw mechanism. For the baseline model, the keypoint detection task holds a larger loss in the early training stage, which dominates backpropagation gradients. This fluctuation between the two tasks forms a typical gradient seesaw pattern, where optimization progress in one task is accompanied by performance regression in the other. For MTC-DETR, both task losses maintain a steady and rapid descent throughout the training process, benefiting from the adaptive uncertainty weighting mechanism of HUDJ-Loss. These visualization results validate the gradient coordination capability of HUDJ-Loss, and confirm that the multi-task collaborative architecture achieves a balanced optimization of heterogeneous tasks.

### 4.7. Failure Modes Analysis

**Figure 14:**
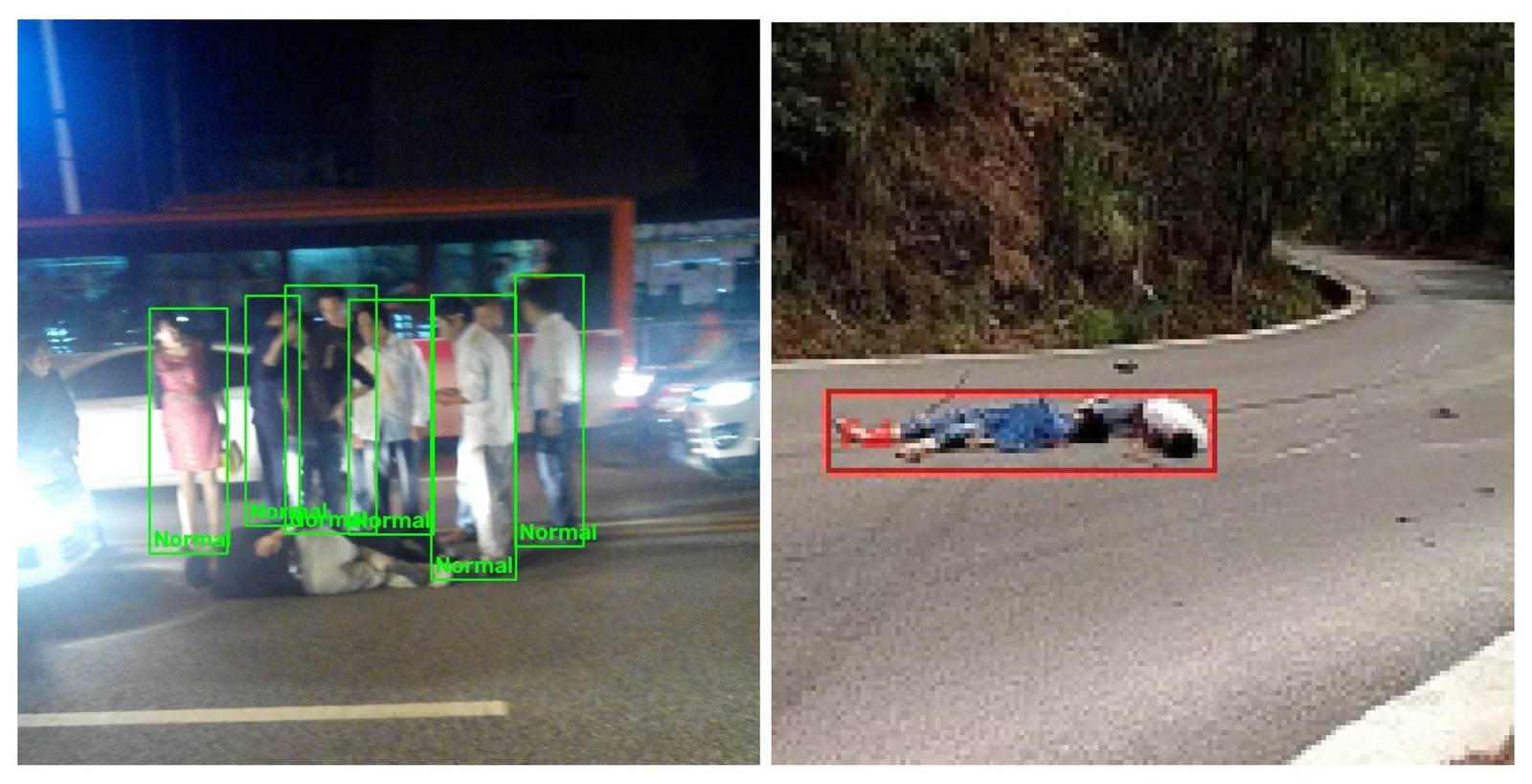
Typical failures in MTC-DETR. Left: missed fall detection under low-illumination night scene; right: instance mis-merging under severe occlusion with low resolution.

To clarify the performance boundaries of the proposed method, two typical failures are visualized in Figure 14.

The first failure is missed detection under extreme low-illumination conditions. Insufficient lighting submerges fine-grained human texture and edge features in image noise, and the graph topology prior cannot compensate for the complete loss. This reflects the inherent limitation of the single RGB modality under extreme lighting environments. The second failure is instance mis-merging in low-resolution scenarios. For falling objects with few effective pixels, inter-person occlusion further blurs the contour boundaries between adjacent instances. Lacking sufficient fine-grained visual features, the model fails to discriminate independent instance boundaries and incorrectly merges two fall objects into a single detection result. Fine-grained instance discrimination under low-resolution occlusion conditions still requires optimization.

### 4.8. Deployment and Quantitative Validation on Real Physical Platforms

**Figure 15:**
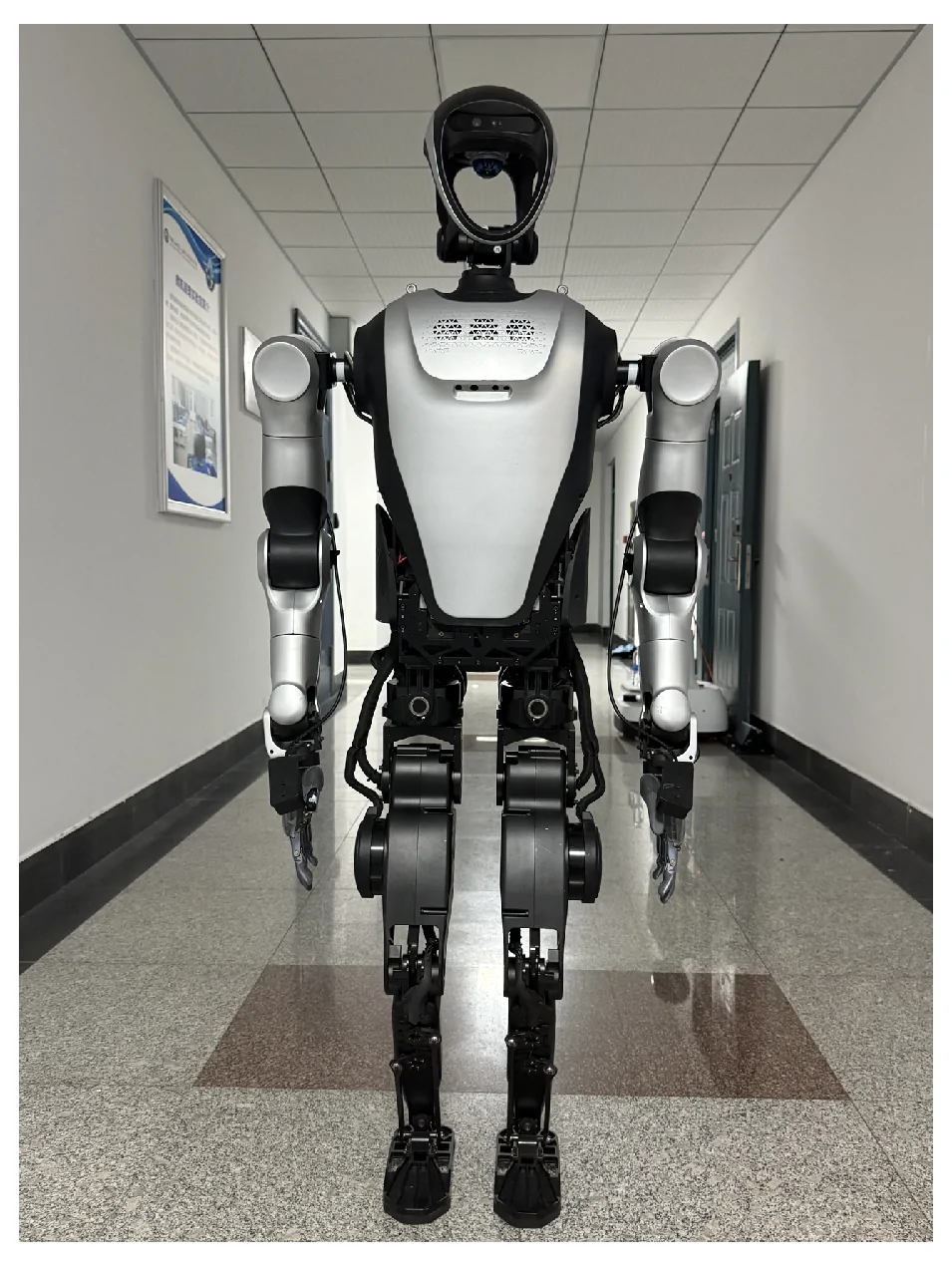
The Leju Kuavo 5 humanoid robot in the experiment.

To verify the practical deployment capability of MTC-DETR on AI edge terminals, where both computational power and power consumption are strictly constrained, edge deployment experiments are conducted based on a Leju Kuavo 5 humanoid robot (manufactured by Shenzhen Leju Robot Technology Co., Ltd., Shenzhen, China) as presented in Figure 15. Its edge computing hardware is the NVIDIA Jetson Orin NX (16GB) module (manufactured by NVIDIA Corporation, Santa Clara, CA, USA). Since the system must process high-load tasks such as gait control and SLAM navigation in parallel, the total power budget allocated to the visual detection system is strictly limited to below 15 W.

#### 4.8.1. Model Quantization and Deployment Pipeline

Two quantization strategies are adopted for edge adaptation, with the full-precision FP32 model as the benchmark. FP16 quantization leverages the native Tensor Core acceleration of the Jetson Orin NX to achieve near-lossless inference and the INT8 full-integer quantization applies an entropy calibration algorithm with 200 randomly sampled images from the training split of the MT-Fall dataset as the calibration set, which shares no overlap with the test samples.

For edge deployment, the original PyTorch model weights are first exported to the ONNX intermediate representation, then optimized and compiled into TensorRT 8.6 inference engines and finally deployed on the robot. All tests are performed under the fixed 15 W low-power mode of the platform.

#### 4.8.2. Performance and Accuracy Loss Analysis

Accuracy metrics and corresponding losses of different quantization strategies are listed in Table 10. Compared with the FP32 baseline, the accuracy loss of the FP16 scheme is controlled within 2%, with negligible impact. The INT8 strategy obtains higher acceleration at the cost of moderate accuracy decline, and the loss range stays within the acceptable threshold of practical scenarios.

The accuracy loss caused by INT8 quantization is a common phenomenon in model edge deployment, which mainly occurs due to three reasons. First, the numerical representation range is compressed from 32-bit floating point to 8-bit integer, which reduces the fineness of feature expression. Low-amplitude discriminative features are easily rounded off during integer mapping, resulting in information loss. Second, when the activation values of feature maps are unevenly distributed with outliers, the unified quantization interval cannot take into account both the main feature range and outlier values, which further amplifies the quantization error of effective features. Third, quantization errors will accumulate layer by layer along the forward propagation of the network, and the deviation in deep features will be gradually enlarged, eventually leading to the decline in overall detection accuracy.

Compensation strategies can be adopted to alleviate the accuracy loss. For example, per-channel quantization can be used to set independent quantization parameters for each feature channel, so as to adapt to different feature distribution characteristics. Quantization-aware training can also be introduced to simulate quantization noise in the training process, so that the model can adapt to integer numerical representation in advance.

In addition, inference performance and resource consumption are summarized in Table 11. Both quantization schemes meet the basic real-time requirement under the 15 W power budget. The INT8 scheme reduces end-to-end latency by about 30% compared with FP16, along with lower memory and power consumption. The two schemes can be selected according to the demand of accuracy priority or energy efficiency priority in actual deployment.

## 5. Conclusions

This paper presents MTC-DETR, a graph topology-guided multi-task collaborative framework for fall detection in complex scenarios. To address the challenges of occlusion and scale variation in multi-person surveillance scenes, the framework designs a Dynamic Scale-Routing and Task-Tuning Encoder, a Topology-Guided Dual-Branch Decoder, and a Homoscedastic Uncertainty Dynamic Joint Loss. Experiments on three datasets demonstrate that the proposed method consistently outperforms 14 state-of-the-art approaches, achieving mAP@0.5 scores of 92.7%, 88.9%, and 90.4%, respectively. Furthermore, the model is deployed on the Leju Kuavo 5 humanoid robot equipped with an NVIDIA Jetson Orin NX edge computing module. With INT8 quantization, the model reaches an inference speed of 44.2 FPS, verifying its practical feasibility for real-time edge fall detection applications.

Despite the performance, the current single-frame static detection paradigm cannot incorporate the temporal dynamic information of the falling process, and there remains an inherent false-positive risk for highly similar static poses such as voluntary lying down. In addition, the current single RGB modality still suffers from performance degradation under low-illumination conditions. Future work will first introduce temporal motion modeling to capture the dynamic characteristics of falls such as rapid center-of-gravity drop and posture mutation, so as to more accurately distinguish accidental falls from non-dangerous active lying behaviors. On this basis, future work will explore multi-modal fusion with infrared thermal imaging or millimeter-wave radar to improve environmental robustness, further extending the application scope of vision-based fall detection systems.

## Figures and Tables

**Table 1:** Detailed statistics of the MT-Fall dataset.

Category	Subcategory	Number	Proportion
Occlusion level	Mild (visible ratio ≥ 80%)	9605	43.0%
	Moderate (80% > visible ratio ≥ 50%)	8642	38.7%
	Severe (50% > visible ratio)	4067	18.2%
Instance scale	Small (pixel area ≤ $32^{2}$)	4729	21.2%
	Medium ($32^{2}$ < pixel area ≤ $96^{2}$)	12,748	57.1%
	Large ($96^{2}$ < pixel area)	4837	21.7%

**Table 2:** Performance comparison of different methods on the CAUCAFall dataset.

Category	Method	mAP@0.5	mAP@0.5:0.95	OKS	Precision	Recall
Object Detection	DETR	82.4	64.5	–	81.3	80.2
	RT-DETR	87.5	70.2	–	85.8	84.6
	DINO-DETR	88.9	72.8	–	87.2	86.4
	YOLO11	87.1	69.8	–	85.0	84.5
	YOLO26	90.3	74.6	–	88.9	88.1
	RT-DETRv4	90.8	75.3	–	89.4	88.5
Keypoint Detection	ViTPose	–	–	86.5	85.8	84.9
	RTMPose	–	–	87.2	86.4	85.6
	ED-Pose	–	–	88.4	87.6	86.8
Multi-Task Learning	HPS-MTL	85.4	68.3	84.6	85.2	84.1
	YOLO-Pose	88.5	73.2	86.9	87.8	86.7
	scMoE	89.3	74.5	87.8	88.5	87.9
	MoDo	90.5	75.8	89.2	89.6	89.2
	Nash-MTL	91.2	76.6	90.1	90.8	90.2
	MTC-DETR	92.7	78.6	91.8	92.5	91.6

**Table 3:** Performance comparison of different methods on the DiverseFall10500 dataset.

Category	Method	mAP@0.5	mAP@0.5:0.95	OKS	Precision	Recall
Object Detection	DETR	78.5	59.8	–	77.2	75.8
	RT-DETR	83.2	64.9	–	81.6	80.5
	DINO-DETR	84.8	66.5	–	83.5	82.2
	YOLO11	82.5	63.2	–	80.4	79.5
	YOLO26	86.8	69.4	–	85.8	84.5
	RT-DETRv4	87.3	70.1	–	86.4	85.1
Keypoint Detection	ViTPose	–	–	81.5	80.6	79.4
	RTMPose	–	–	82.4	81.3	80.2
	ED-Pose	–	–	83.9	82.7	81.5
Multi-task Learning	HPS-MTL	80.2	61.5	80.4	79.8	78.5
	YOLO-Pose	84.1	66.8	82.6	83.2	82.0
	scMoE	85.5	68.4	84.1	84.6	83.8
	MoDo	86.7	70.2	85.8	86.2	85.4
	Nash-MTL	87.5	71.5	86.9	87.4	86.6
	MTC-DETR	88.9	74.3	89.5	89.8	88.9

**Table 4:** Performance comparison of different methods on the MT-Fall dataset.

Category	Method	mAP@0.5	mAP@0.5:0.95	OKS	Precision	Recall
Object Detection	DETR	73.2	53.4	–	71.5	69.8
	RT-DETR	79.4	58.6	–	77.8	75.4
	DINO-DETR	81.6	61.2	–	80.2	78.5
	YOLO11	78.5	57.1	–	76.5	74.2
	YOLO26	84.1	64.8	–	82.6	80.9
	RT-DETRv4	84.8	65.5	–	83.2	81.5
Keypoint Detection	ViTPose	–	–	74.6	73.8	71.5
	RTMPose	–	–	75.8	74.6	72.8
	ED-Pose	–	–	78.2	77.5	75.4
Multi-Task Learning	HPS-MTL	77.4	56.8	76.5	76.1	74.6
	YOLO-Pose	80.8	61.4	78.9	79.6	78.2
	scMoE	82.6	63.8	80.5	81.8	80.4
	MoDo	84.5	66.4	82.4	83.9	82.8
	Nash-MTL	85.8	68.2	84.6	85.5	84.2
	MTC-DETR	90.4	75.9	89.3	91.4	90.6

**Table 5:** Inference efficiency and complexity comparison on the MT-Fall dataset.

Method	Params (M)	FLOPs (G)	FPS	mAP@0.5 (%)
DETR	41.3	90.0	22.4	73.2
DINO-DETR	47.0	170.0	38.1	81.6
RT-DETR	24.8	32.6	112.5	79.4
RT-DETRv4	24.8	32.6	108.3	84.8
YOLO11	56.9	194.9	68.2	78.5
YOLO26	68.9	205.6	62.7	84.1
YOLO-Pose	56.9	194.9	65.4	80.8
HPS-MTL	26.2	35.1	95.6	77.4
scMoE	27.5	36.8	83.7	82.6
MoDo	26.2	35.1	93.8	84.5
Nash-MTL	26.2	35.1	94.7	85.8
MTC-DETR (Ours)	29.5	41.3	86.3	90.4

**Table 6:** Ablation results of core components on the MT-Fall dataset.

Components	Modules	mAP@0.5	mAP@0.5:0.95	OKS	Precision	Recall
Baseline	RT-DETR	78.1	57.0	76.3	76.5	74.3
Encoder	+ DLKA	80.8	59.8	78.1	79.2	77.0
	+ DCF-TAD	83.4	63.0	80.0	81.8	79.7
Decoder	+ TG-LGSA	85.0	65.8	83.5	84.3	82.6
	+ Pose Prior Injection	87.6	71.5	87.2	88.5	87.2
Loss	+ HUDJ-Loss	90.4	75.9	89.3	91.4	90.6

**Table 7:** Incremental ablation results of TG-LGSA components on the MT-Fall dataset.

Configuration	mAP@0.5	OKS	Precision	Recall
Baseline (vanilla self-attention)	83.4	80.0	81.8	79.7
+ multi-hop GCN	84.5	82.6	83.5	81.7
+ TPE	84.6	82.9	83.7	82.0
+ SPD	84.7	83.3	83.9	82.1
+ adaptive gated fusion (full TG-LGSA)	85.0	83.5	84.3	82.6

**Table 8:** Comparison of different multi-task loss strategies on the MT-Fall dataset.

Loss Strategy	mAP@0.5	mAP@0.5:0.95	OKS	Precision	Recall
Equal Weight	86.2	69.8	85.8	87.1	85.7
Fixed Weight (baseline)	87.6	71.5	87.2	88.5	87.2
GradNorm	88.5	72.8	88.1	89.2	88.0
DWA	88.9	73.4	88.4	89.6	88.3
HUDJ-Loss (Ours)	90.4	75.9	89.3	91.4	90.6

**Table 9:** Performance comparison under different occlusion severity levels on the MT-Fall dataset.

Method	Metric	Mild	Moderate	Severe
RT-DETR	mAP@0.5	85.2	76.8	63.1
	OKS	–	–	–
YOLO-Pose	mAP@0.5	86.9	79.3	68.5
	OKS	83.2	78.5	71.4
Nash-MTL	mAP@0.5	90.1	84.7	75.8
	OKS	88.5	84.2	78.3
**MTC-DETR**	mAP@0.5	93.8	90.2	84.6
	OKS	92.7	89.1	85.2

**Table 10:** Accuracy and loss comparison of different quantization schemes.

Quantization Mode	mAP@0.5 (%)	OKS (%)	mAP Loss (%)	OKS Loss (%)
FP32 (Desktop Baseline)	90.4	89.3	0.0	0.0
FP16 (Edge)	88.7	87.9	1.7	1.4
INT8 (Edge)	86.2	85.5	4.2	3.8

**Table 11:** Inference performance and resource consumption on edge platform.

Quantization Mode	Inference FPS	Latency (ms)	Memory (MB)	Average Power (W)
FP16 (Edge)	27.6	47.8	582	10.7
INT8 (Edge)	44.2	33.5	327	7.9

## Data Availability

The CAUCAFall dataset and DiverseFall10500 dataset are publicly available benchmarks. The CAUCAFall dataset can be obtained from the work of Guerrero et al. [16], and the DiverseFall10500 dataset is introduced in the research of Khan et al. [17]. The raw data of the MT-Fall dataset used in this study is publicly available at https://github.com/PixelHike/MT-Fall-dataset (accessed on 22 June 2026).
